# What evidence exists on wild bee trends in Germany? A systematic map

**DOI:** 10.1186/s13750-025-00364-7

**Published:** 2025-06-19

**Authors:** Anne-Christine Mupepele, Niels Hellwig, Petra Dieker, Alexandra-Maria Klein

**Affiliations:** 1 Animal Ecology, Department of Biology, University of Marburg, Karl-von-Frisch-Straße 8, 35043 Marburg, Germany; 2 Thünen Institute of Biodiversity, Bundesallee 65, 38116 Braunschweig, Germany; 3 Department of Agriculture, Ecotrophology and Landscape Development, Anhalt University of Applied Sciences, Strenzfelder Allee 28, 06406 Bernburg (Saale), Germany; 4 Agroecology and Environment, Agroscope, Reckenholzstrasse 191, Zürich, 8046 Switzerland; 5 Thünen Institute of Farm Economics, Bundesallee 63, 38116 Braunschweig, Germany; 6 Nature Conservation and Landscape Ecology, University of Freiburg, Stefan-Meier Straße 76, 79111 Freiburg, Germany

**Keywords:** Biodiversity loss, Hymenoptera, Conservation, Population trends, Landscape ecology

## Abstract

**Background:**

Wild bees have attracted growing attention from both the scientific community and civil society, alongside increasing evidence of biodiversity losses. Declining wild bee populations threaten both the quality and quantity of pollination, which also affect crop production and are therefore critically important for human wellbeing. Landscape homogenisation, land use changes, land use intensity, and climate change are driving the decline. Despite concerns about the wild bee decline, knowledge of wild bee population patterns and long-term trends across Germany remains limited. Here, we present a systematic map, including a newly developed comprehensive database that compiles available data on temporal trends in wild bee communities across Germany. Our goal is to provide an overview of the frequency of wild bee trend studies over time and the land use types and geographical areas they have covered.

**Methods:**

Our search for data on wild bee trends was conducted in November 2020 and included peer-reviewed literature (from Web of Science databases and Scopus) and grey literature in English and in German. After screening the literature by title and abstract, relevant data were extracted from eligible studies. All eligible studies included data on wild bee taxa from at least two years at the same site within Germany and using the same sampling method. The database consists of data sheets on studies (bibliographic context), on covariates (methodological and spatio-temporal context) and on data (bee species sampled at a specific time on a specific location – exemplarily for two German regions).

**Review findings:**

The database contains 382 studies out of 24,486 initial records. Nearly 75% of the full texts screened did not include field data on wild bees from at least two different years and were therefore excluded. Studies date back to the 1880s, with a consistently high number of studies since the 1990s. Most studies were published in German-language journals of entomological societies in Germany. Data originate from different types of land use throughout the country, mostly from southern and north-western Germany and from urban areas.

**Conclusions:**

The systematic map shows that there is a lack of long-term monitoring studies. Moreover, there are research gaps in land use categories and federal states, which hinder more regional or land use-specific analyses. We encourage researchers and practitioners to use the database for further analyses on bee trends and their drivers, and the potential success of mitigation measures.

**Supplementary Information:**

The online version contains supplementary material available at 10.1186/s13750-025-00364-7.

## Background

Wild bees have long inspired humans with their beautiful appearance, and their benefits in plant pollination [1]. They have attracted widespread public attention and have been the focus of German petition campaigns to reverse insect decline [2–5]. Scientists have intensively studied wild bees, and particularly their role as pollinators, in recent decades [6–11].

The decline of wild bees and pollinators in general has been documented worldwide [12–15] and is in line with the overall biodiversity loss [16, 17]. Given their role as pollinators for crops in particular, the decline of wild bees may also affect economic production [18–21].

The decline of wild bees is often linked to changes in air temperature, precipitation, landscape structure (composition and configuration, small landscape features), land use changes, and land use intensity (pesticides, fertiliser, mowing frequency in agricultural areas; tree cover density in forests; impervious surface density in urban areas) [22–26]. Especially land use and landscape heterogeneity have a strong influence on changes in wild bee populations, with degradation causing declines [27]. It is thus essential to identify which landscape and land use changes are occurring and how structural elements contribute to the conservation of wild bee populations.

Trends in wild bees have been analysed on large scales based on data from local-scale monitoring activities or museum collections (e.g [13, 14, 28, 29]). Approximately 600 wild bee species have been described for Germany, with 232 species categorized at least as endangered in the latest national Red List of wild bees published in 2011 [11, 30, 31]. Moreover, the recent political debate on declining insect populations began in Germany [16, 30], leading to initiatives for standardized surveys of wild bees and other flower visitors (e.g [31, 32]). However, data remains scarce, and knowledge of German wild bee communities is fragmented. During a 2020 workshop with German stakeholders—part of the sMon project (“Analysing trends in German species data”) led by the German Centre for Integrative Biodiversity Research in Halle, Jena, and Leipzig—stakeholders and researchers identified the need to improve knowledge on German bees. We proposed the idea of creating a systematic map through an intensive search, recognizing that various entomological societies, nature conservation authorities across all 16 federal states, and several independent wild bee experts likely hold valuable records on wild bees. Collecting and synthesising this information is a laborious process, but it holds great potential to significantly enhance our understanding of wild bee population trends in Germany, offering a foundation for future decisions on pollinator conservation—even at the EU or global level [3]. Using Germany as an example, we further demonstrate the benefits of searching grey literature in the local language [32].

## Objective of the review

The scope of this systematic map is to introduce a new comprehensive database that will allow the identification of trends in wild bee populations in Germany. Therefore, we aim to identify available data on temporal trends of wild bee communities, mainly in agricultural areas, but also in urban areas and forests. As outlined in our protocol, the database contains information extracted from studies on trends in wild bees [33]. We expect trends to differ according to land use types and legal regulations in the federal states.

According to our protocol, we aim to identify whether diversity has changed over time and whether diversity trends differ between landscape types (agricultural, forest, urban) and federal states. Given the number of studies we have found in the grey literature, e.g., literature not covered by Web of Science and Scopus searches and usually not peer-reviewed, extracting data to analyse trends takes considerably more time than expected. We are therefore keen to share the database with scientists to enable further analyses and maximise its potential, especially because wild bee trends are a timely topic requested by nature conservationists, policy makers, and scientists. The current systematic map answers the questions: How many studies have looked at changes in diversity over time (1), and what land use types and federal states have these studies covered (2).

The systematic map results will be used by scientists to further analyse the causes and consequences of changes in wild bee population. Practitioners working on conservation of wild bees can use the database to derive information on specific species and land use to identify suitable habitat types and conservation measures, particularly in Germany, but also across Europe.

## Methods

The methods of this systematic map were first described in our protocol on “osf” [34] and afterwards updated and further developed in our protocol on “PROCEED” [33]. All methods are based on the ROSES reporting standards and guidelines of the Collaboration for Environmental Evidence (version 5.1) [35, 36].

### Deviations from the protocol

Due to the large number of extracted studies, we restricted the objective of the review to the identification of studies on wild bee trends together with their bibliographic, methodological, and spatio-temporal context. All this information was included in the originally planned database tables of studies and covariates [33]. However, due to limited resources, it was not possible to extract detailed data on wild bee diversity and abundance (e.g., numbers of species and individuals per location and time) from all sources. As an example for the potential use of the database, we include detailed data on wild bees for two German regions: Hesse and Saxony-Anhalt.

### Search for articles

Our search comprised German and English data sources on the diversity and abundance of wild bees and was conducted in November 2020. Several German-language journals report on the conservation status of wild bees in grey literature. Therefore, we intensively searched both peer-reviewed literature (see list below: bullet Points 1–2) and grey literature (see list below: bullet Points 3–7).

We searched for literature in the following sources:

1. Published and peer-reviewed literature from bibliographic databases.
2. Peer-reviewed journals publishing in German: ‘Natur und Landschaft’.
3. Contributions and German-language journals from entomological societies in Germany (see [34])
4. References from the book: ‘Wildbienen Deutschlands’ [37].
5. Public authorities (‘Behörden’).
6. Contacting German wild bee specialists and our stakeholders (i.e., researchers and experts attending a workshop at the German Centre for Integrative Biodiversity Research in 2020).
7. Databases and data repositories.

We searched published and peer-reviewed literature from the Web of Science and Scopus using a search string that covered potential data sources (see [34]). The Web of Science search covered the following databases: the Web of Science Core Collection, Biological Abstracts, CAB Abstracts, KCI-Korea Journal Database, Russian Science Citation Index, Scielo Citation Index, BIOSIS Citation Index, BIOSIS Previews, Current Contents Connect, Data Citation Index, Derwent Innovations Index, MEDLINE (R), Zoological Record. We only searched for studies that either included the term “Germany” or one of the federal states in the keywords, title, or abstract, or had at least one co-author affiliated with a German institution. The Web of Science and Scopus searches were conducted as topic searches with the following search string (see also additional file S1):

(TS=(German* OR Deutsch* OR “Baden-Wuerttemberg” OR “Baden-Württemberg$” OR Bavaria OR Bayern$ OR Berlin$ OR Brandenburg$ OR Bremen$ OR Hamburg$ OR Hesse OR Hessen$ OR “Mecklenburg-Western Pomerania” OR “Mecklenburg-Vorpommern$” OR “Lower Saxony” OR Niedersachsen$ OR “North Rhine-Westphalia” OR “Nordrhein-Westfalen$” OR “Rhineland-Palatinate” OR “Rheinland-Pfalz” OR Saarland* OR Saxony OR Sachsen$ OR “Saxony-Anhalt” OR “Sachsen-Anhalt$” OR “Schleswig-Holstein$” OR Thuringia OR Thüringen$) OR AD=(Germany OR Deutschland OR “Baden-Wuerttemberg” OR “Baden-Württemberg” OR Bavaria OR Bayern OR Berlin OR Brandenburg OR Bremen OR Hamburg OR Hesse OR Hessen OR “Mecklenburg-Western Pomerania” OR “Mecklenburg-Vorpommern” OR “Lower Saxony” OR Niedersachsen OR “North Rhine-Westphalia” OR “Nordrhein-Westfalen” OR “Rhineland-Palatinate” OR “Rheinland-Pfalz” OR Saarland OR Saxony OR Sachsen OR “Saxony-Anhalt” OR “Sachsen-Anhalt” OR “Schleswig-Holstein” OR Thuringia OR Thüringen)) AND TS=(“wild bee$” OR “trap nesting bee$” OR “cavity nesting bee$” OR “ground nesting bee$” OR “solitary bee$” OR Anthophila OR Apiformes OR Apoidea OR Andrenid* OR Apid* OR Colletid* OR Halictid* OR Megachilid* OR Melittid* OR Ammobates OR Ammobatoides OR Andrena OR Anthidium OR Anthophora OR Biastes OR Bombus OR Camptopoeum OR Ceratina OR Chelostoma OR Coelioxys OR Colletes OR Dasypoda OR Dioxys OR Dufourea OR Epeoloides OR Epeolus OR Eucera OR Halictus OR Heriades OR Hylaeus OR Lasioglossum OR Lithurgus OR Macropis OR Megachile OR Melecta OR Melitta OR Melitturga OR Nomada OR Nomia OR Nomioides OR Osmia OR Panurginus OR Panurgus OR Rhophitoides OR Rophites OR Sphecodes OR Stelis OR Systropha OR Thyreus OR Xylocopa OR “mining bee$” OR “carder bee$” OR “potter bee$” OR “flower bee$” OR “digger bee$” OR bumblebee$ OR “bumble bee$” OR “carpenter bee$” OR “scissor bee$” OR “sharp-tail bee$” OR “plasterer bee$” OR “vernal bee$” OR “ivy bee$” OR “sea aster bee$” OR “hairy-legged bee$” OR “pantaloon bee$” OR “long-horned bee$” OR “furrow bee$” OR “resin bee$” OR “yellow-faced bee$” OR “yellow-face bee$” OR “yellow loosestrife bee$” OR “leafcutter bee$” OR “leaf-cutter bee$” OR “leafcutting bee$” OR “leaf-cutting bee$” OR “sainfoin bee$” OR “red bartisia bee$” OR “nomad bee$” OR “mason bee$” OR “shaggy bee$” OR “gray-haired bee$” OR “alfalfa bee$” OR “blood bee$” OR “dark bee$” OR “spiral-horned bee$” OR “cuckoo bee$” OR pollinat* OR Wildbiene$ OR Sandgängerbiene$ OR Steppenglanzbiene$ OR Sandbiene$ OR Wollbiene$ OR Harzbiene$ OR Pelzbiene$ OR Kraftbiene$ OR Hummel$ OR Buntbiene$ OR Keulhornbiene$ OR Scherenbiene$ OR Kegelbiene$ OR Seidenbiene$ OR Hosenbiene$ OR Zweizahnbiene$ OR Glanzbiene$ OR Schmuckbiene$ OR Filzbiene$ OR Langhornbiene$ OR Furchenbiene$ OR Löcherbiene$ OR Maskenbiene$ OR Schmalbiene$ OR Steinbiene$ OR Schenkelbiene$ OR Blattschneiderbiene$ OR Mörtelbiene$ OR Trauerbiene$ OR Sägehornbiene$ OR Schwebebiene$ OR Wespenbiene$ OR Schienenbiene$ OR Steppenbiene$ OR Mauerbiene$ OR Scheinlappenbiene$ OR Zottelbiene$ OR Graubiene$ OR Schlürfbiene$ OR Buckelbiene$ OR Düsterbiene$ OR Spiralhornbiene$ OR Fleckenbiene$ OR Holzbiene$ OR Bestäub*).

We have accessed the Web of Science and Scopus via the subscriptions of the University of Freiburg and the Thünen Institute Braunschweig. As we focused our review on Germany, we had to restrict the search terms accordingly. Based on our knowledge and feedback from our stakeholders (i.e., researchers and experts attending a workshop at the German Centre for Integrative Biodiversity Research in 2020), we did not expect to find additional studies relevant to our review. For the grey literature search, we screened the content of 75 journals of entomological societies in Germany [34]. The search included articles published up to November 2020.

To assess the comprehensiveness of the search, we used four benchmark publications, i.e., publications relevant for our systematic map [38–41]. These benchmark publications were provided by our pre-analysis protocols [33, 34]. All benchmark publications were identified by the search string used in the Web of Science and Scopus searches, demonstrating the suitability of the search string. Moreover, a web-based scoping search, using the keywords: “bee AND Germany AND trend”, revealed no additional studies. As a result, we concluded that the literature sources included in our systematic map already encompassed all relevant grey and peer-reviewed sources. Therefore, additional web-based search engines were not included in the final search.

Contacts with nature conservation authorities from local to federal level, wild bee specialists, and our stakeholders (see above) did not provide additional data on wild bee diversity and abundance trends. Databases and data repositories included the Global Biodiversity Information Facility [42] and the Conservation Evidence Synopses [43]. German entomological societies occasionally provided data, but this would have been additionally published in reports and annual reviews which we covered in bullet Point 3.

### Screening process

We first screened all literature for title and abstract. If the title and abstract did not allow exclusion based on one of the non-fulfilled eligibility criteria, we read the full text. We then extracted all relevant information from the full text directly into an SQL database (MariaDB).

We performed a test for consistency of study inclusion between researchers at title and abstract level (unweighted Cohen’s Kappa with higher and lower bound = 0.78 [0.59;0.99]. This reflects 94% of agreement prior resolving inconsistencies [44]). All researchers involved in the screening assessed the first 100 studies of the list extracted from the bibliographic databases, in random order of studies. The screening results were compared with regard to the inclusion or exclusion of studies based on the eligibility criteria, and any disagreements were discussed. In cases of disagreement, the researchers identified the reasons behind one reviewer’s exclusion of a study and the others’ decision to include it. Through discussion, they reached a mutual understanding of the specific exclusion criterion and subsequently revised the description of the exclusion criteria to enhance clarity and consistency for the remaining screening process. Importantly, researchers involved in this publication did not screen or assess any studies in which they were involved themselves.

## Eligibility criteria

The eligibility criteria and formulation of the research question follow the PerSPECTiF framework [45]:

- Perspective: From the perspective of wild bees: Wild bees, i.e., Hymenoptera of the families Apidae (excluding the domestic honeybees), Megachilidae, Halictidae, Colletidae, Andrenidae, Melittidae [46].
- Setting: Covering the geographical scope of Germany in its administrative boundaries in 2020.
- Phenomenon of interest/problem: Trends in German wild bee populations.
- Environment: Samples from all major German landscape types – such as grasslands, urban areas, and forests – are included. All sampling activities must be documented transparently.
- Temporal scale: Data from at least two either consecutive or more distant years at the same site and with the same sampling protocol, i.e., method, are required. At least two consecutive years were selected to enable drawing a regression line, which might be unreliable based on individual studies alone but becomes more meaningful when used in synthesis [47].
- Findings: Outcome measure/data requirements: Occurrence as presence-only, presence-absence, or abundance, but no model or opinion-based data.

This results in the following inclusion criteria that had to be met for a study or dataset to be included in our database:

- Population: Wild bees, i.e., Hymenoptera of the families Apidae (excluding the domestic honeybees), Megachilidae, Halictidae, Colletidae, Andrenidae, Melittidae [46].
- Geographical scope: Germany in its administrative boundaries in 2020.
- Outcome measure/data requirements: Occurrence as presence-only, presence-absence, or abundance, but no model or opinion-based data.
- Temporal scale: Data from at least two either consecutive or more distant years at the same site and with the same sampling protocol, i.e., method, are required.
- Study designs: Observational and experimental data on presence or abundance are included.
- Sampling methods: All approaches (e.g., transect walks or plant observations) are included, but must be consistent across years in a dataset and documented transparently.

## Study validity assessment

No formal study validity assessment has been conducted. The studies are not primarily used for their findings, but rather for their data, such as records of bee occurrences and abundance. Additional information that may inform a future assessment of data quality – such as sampling methods or study design – has been extracted and is presented in the covariates table.

### Data coding strategy

Data extracted from the full texts was entered directly into a database using a custom form developed with R Shiny (version 1.5.0) [48]. The database consisted of data sheets on studies (bibliographic context) and on covariates (methodological and spatiotemporal context). All information relevant to a “bibtex” entry, such as study type, authors, title, and year of publication, was entered into the Study Table. Information on the geographical and methodological context of the studies was entered into the Covariate Table with a link to the corresponding entry in the Study Table. This information included the taxonomic group covered (i.e., species, genus, family), the federal state, the land use, information on the sampling frequency, methods, time period covered, and measure (e.g., biweekly net sweeps from April to August named/reported as abundance per species), and the start and end years of data collection. Additionally, we extracted wild bee data from two German regions: Hesse and Saxony-Anhalt into a preliminary Data Table. Consistency of data coding of the reviewers was verified by the two first authors who evaluated the first data entries of every reviewer involved in the data coding. If data were missing or unclear, we used internet search engines, such as www.ecosia.org, to identify contact information of authors and contacted them asking for additional information.

### Data mapping

We mapped the database through descriptive analyses of the bibliographic, methodological, and spatio-temporal context of the studies, as included in the Study Table and Covariate Table. Evidence was synthesised in tables, graphs, and maps, including descriptive statistics on, for example, the taxonomic groups, sampling methods, locations, land use context, and years covered by the studies. Figures were created in R version 4.2.3 (2023-03-15) [49] and maps were generated in QGIS (version 3.34.3) [50]. Using descriptive figures such as bar plots and box plots, we illustrate the regions where most studies have been conducted and highlight areas where significant knowledge gaps remain.

### Review findings

**Fig. 1:**
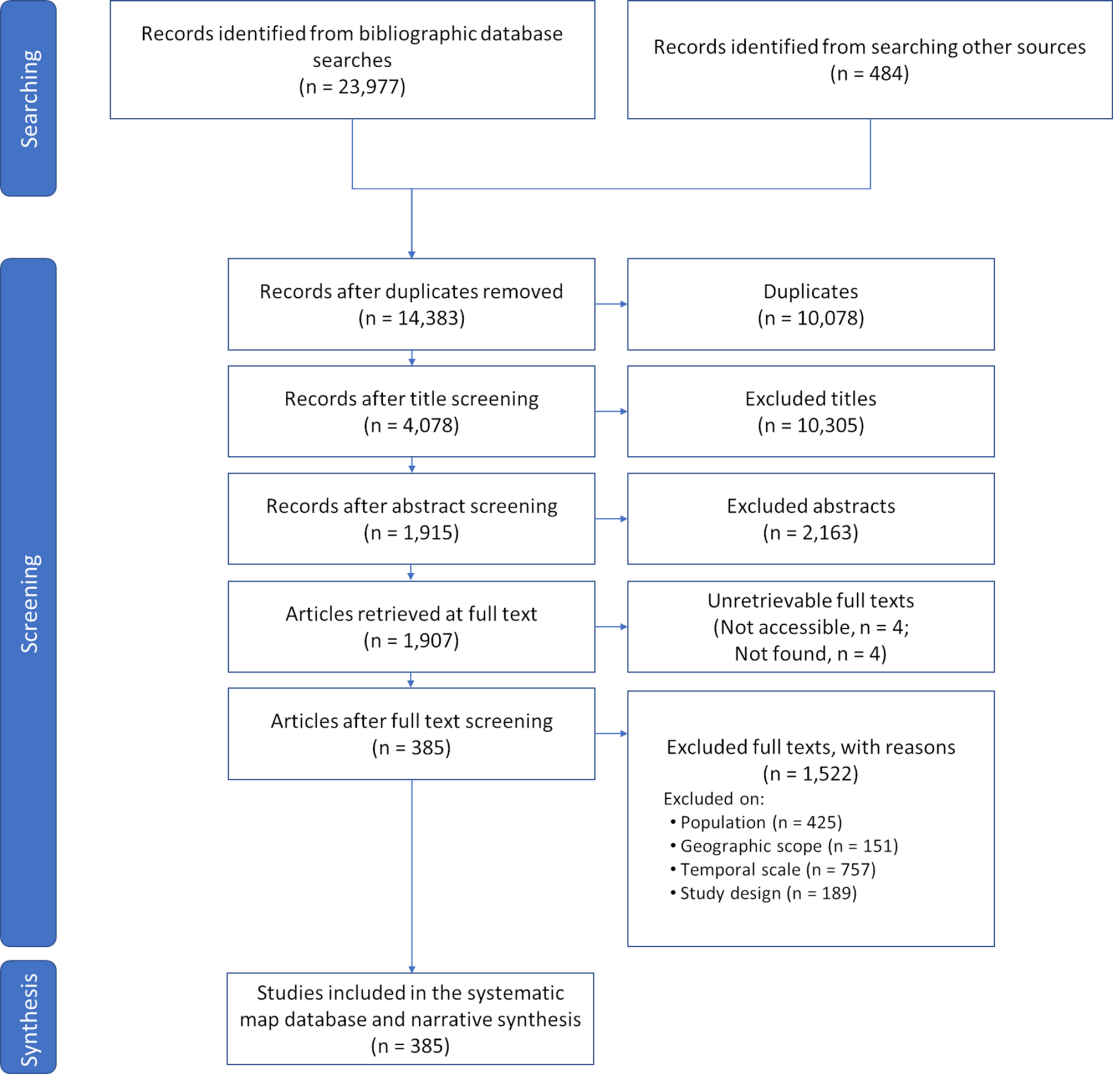
ROSES diagram (according to Haddaway et al. [36]). Reasons for excluded full texts refer to the eligibility criteria as mentioned in the Methods section

In this comprehensive literature search on German wild bee trends, we compiled a database of 385 studies from 24,461 initial records (Fig. 1, see also additional files S2, S3, and S4). Almost half of these initial records were duplicates, which was mostly due to a considerable overlap in the results of the Web of Science and Scopus searches. After screening titles and abstracts, we searched for the full text of the articles and we were able to retrieve 99.6% of them. Only 8 articles could not be found or accessed (reason for exclusion coded as “nAccess”, additional files S5 and S6). 79.8% of all full texts had to be excluded, mostly because they did not include any field data on wild bees (excluded on population, “nPop”) or data from at least two different years (excluded on temporal scale, “nTime”). Moreover, a considerable number of full texts were excluded on study design (“nDesign”) or on geographic scope (“nGeo”).

Some of the included studies had a common study context, i.e., they used the same data, continued data, or complementary data. These clusters of studies were listed in additional file S7. As a result of using the same data, some of these clustered studies were associated with the same covariate entry (see information on covariates in additional file S7).

### Studies on wild bee diversity trends (Study Table)

**Fig. 2:**
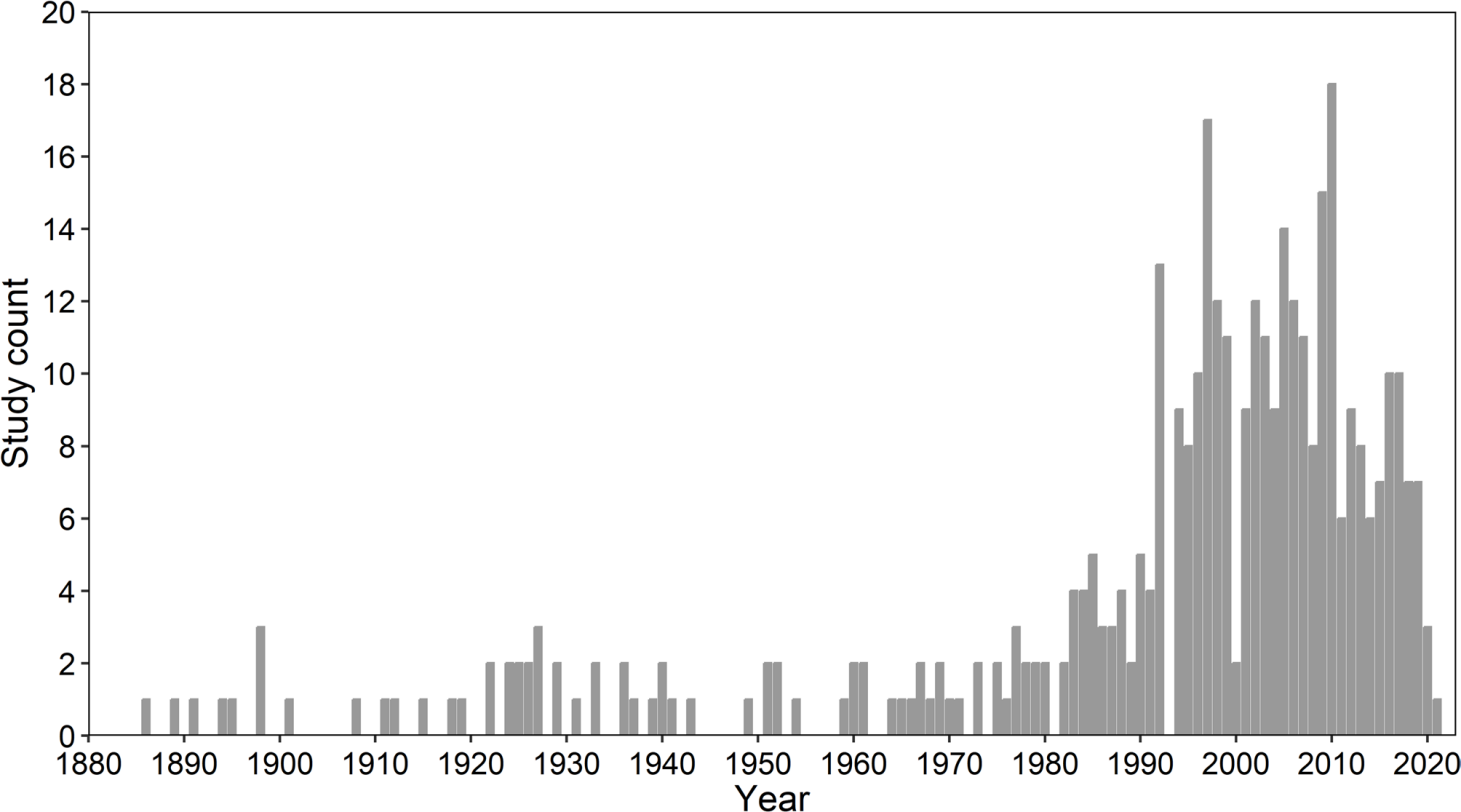
The number of studies per year of publication (see Study Table)

There was a consistently high number of studies between 1990 and 2020. This was followed by an increase in the number of studies in the second half of the 20th century, a pattern similar to that found in other reviews (see for example [51]). We found a low, but constant number of records for the last 100 years, which is promising for the analysis of bee trends and their drivers in Germany (Fig. 2).

**Fig. 3:**
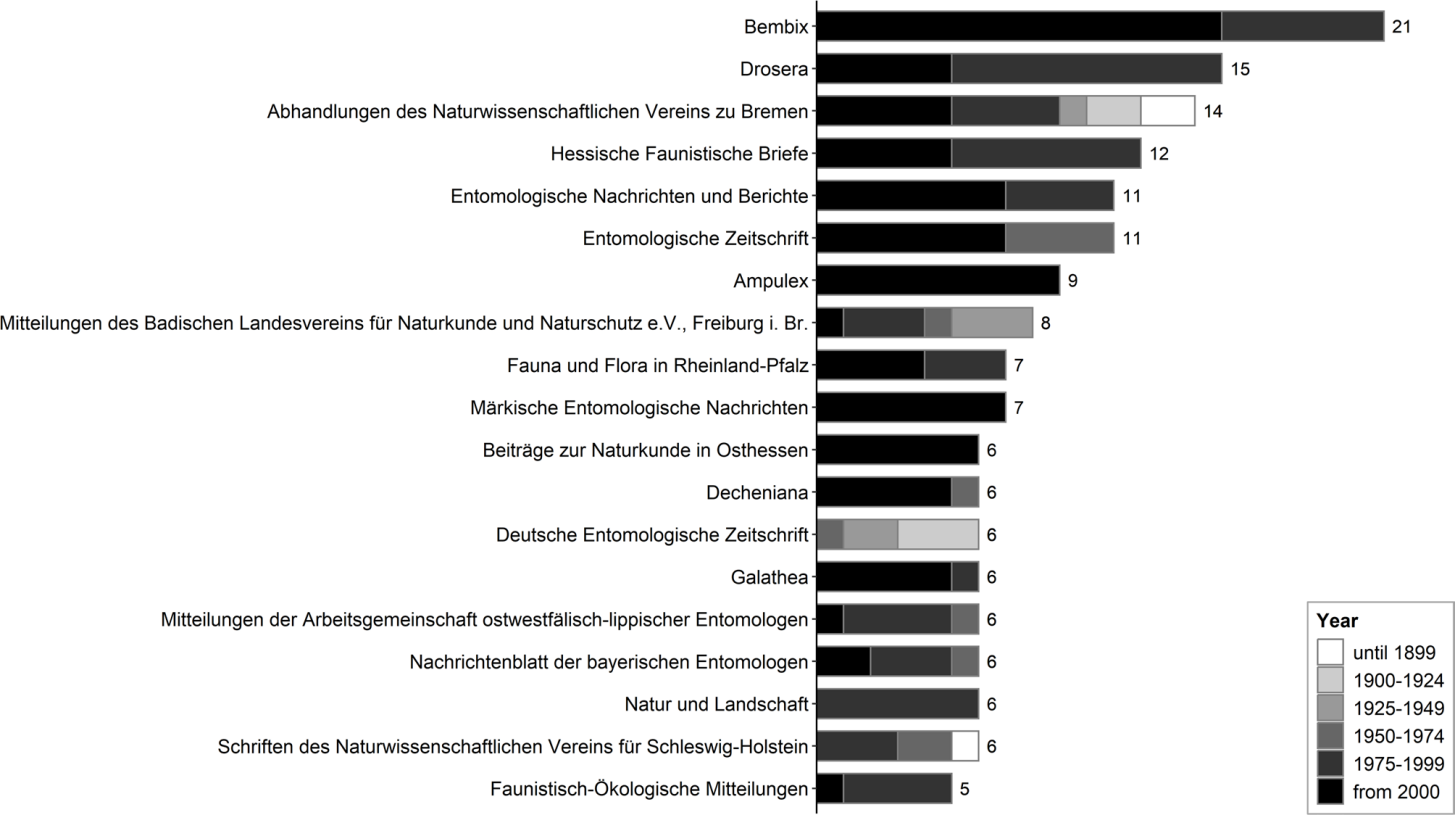
Number of studies per journal, classified into years of publication. The figure includes all journals with minimum five studies covered in the Study Table

Of all the studies included in the Study Table, most were published in German-language journals from entomological societies in Germany. The main outlets for older studies from the 19th century and the first half of the 20th century were *Abhandlungen des Naturwissenschaftlichen Vereins zu Bremen*, *Mitteilungen des Badischen Landesvereins für Naturkunde und Naturschutz e. V.*,* Freiburg i. Br.*, and *Deutsche Entomologische Zeitschrift*. Studies from the second half of the 20th century and the beginning of the 21st century were frequently published in *Bembix*, *Drosera*, and several other German-language journals (Fig. 3).

### Spatio-temporal and methodological study context (Covariate Table)

The Covariate Table contains an entry (row) for each site-study combination (with two sites being separated by at least ~ 20 km). We added further entries if a study investigated only a few bee species or a particular genus, such as *Bombus*, creating species/genus-site-study combinations. For every entry, we extracted bee species-specific, site-specific, and time-specific information. Bee species information was recorded in four columns, including species name, genus, family, and their nesting guild (cavity-nesting, ground-nesting, or parasitic). In most cases, several bee species were studied and sampled with more comprehensive approaches, in which case we entered ‘multiple’ in the database. The site-specific information included the federal state of Germany where the data were collected, the name of the site, the dominant land use type, and geographic coordinates if available. Sample-specific information was classified into sampling time with start and end date, sampling intensity, sampling method, and the output variable, such as presence-absence or abundance data.

**Fig. 4:**
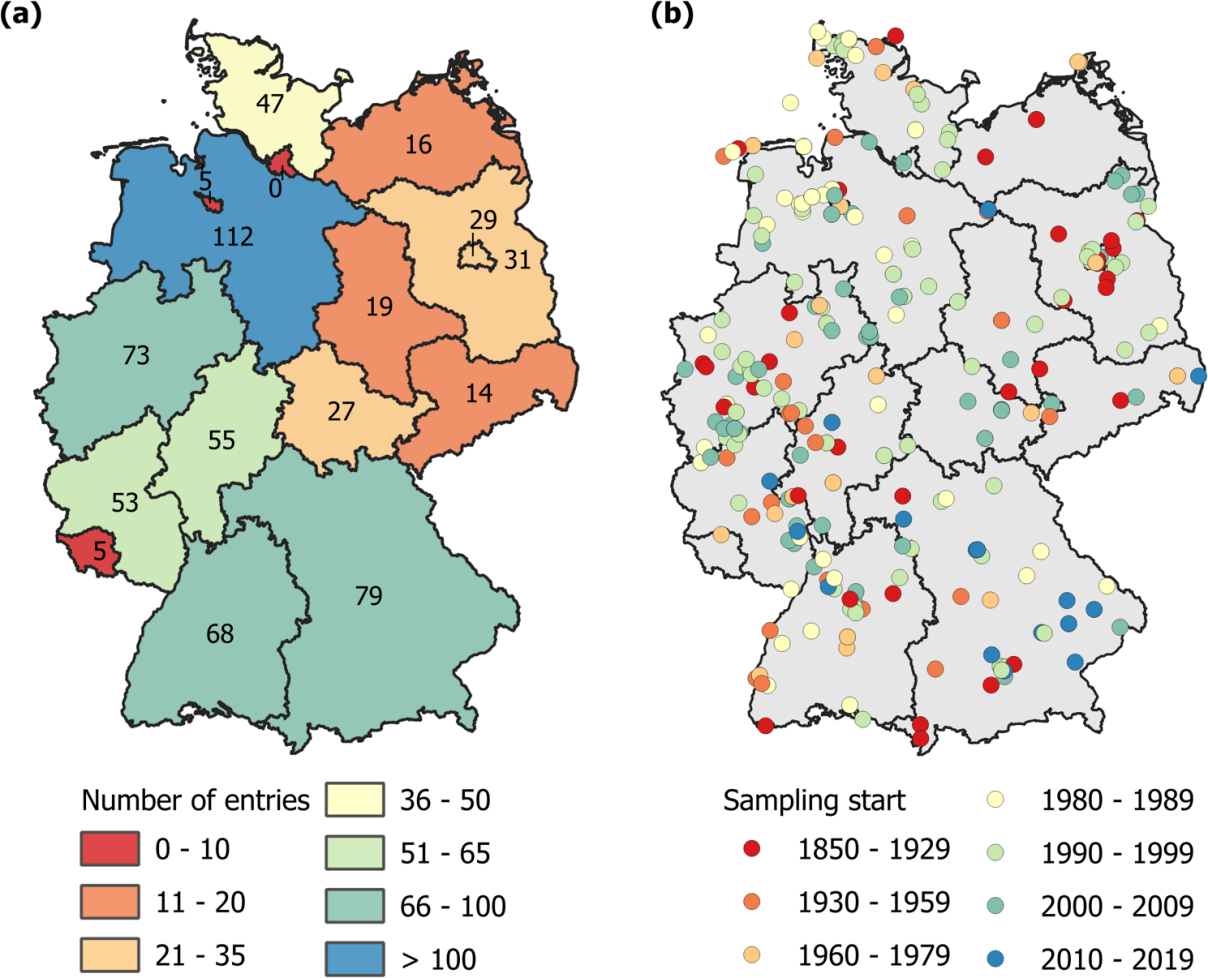
Spatial distribution of entries covered in the Covariate Table: (**a**) database entries per federal state and (**b**) locations from area and site information (as identifiable from 313 out of 633 entries)

**Fig. 5:**
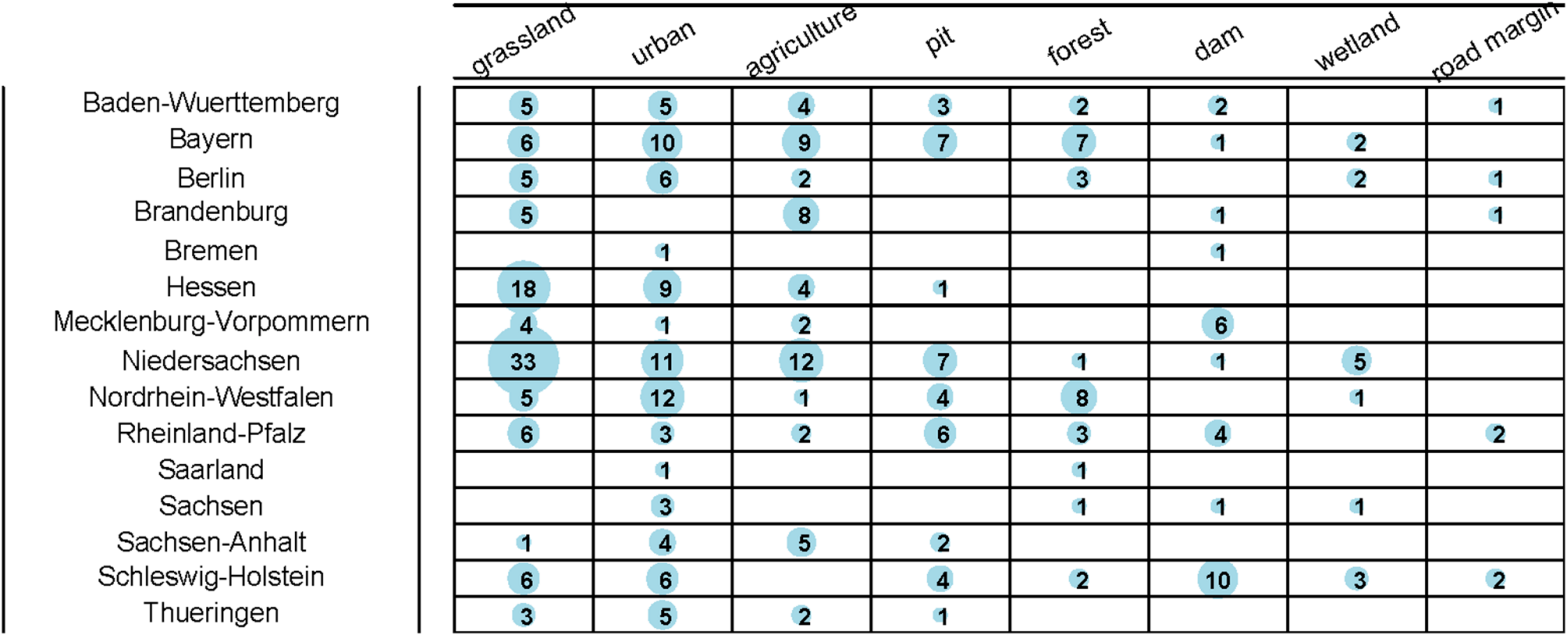
The land use categories surveyed in each federal state showed a good coverage of land use categories – especially grassland and urban areas – across Germany

This information allowed us to identify local differences in studies with respect to German federal states and land use types (Figs. 4 and 5). The studies in our database covered all of Germany (Fig. 4a). Most records came from Lower Saxony in the northwest, followed by Bavaria in the south. The lowest number of records were obtained from the city-states Hamburg and Bremen as well as of the smallest federal state (Saarland). Our database included records from the 19th century to 2020 without any systematic spatio-temporal correlation (Fig. 4b). This means that we included data from all over Germany in all time periods.

Different types of land use were sampled across Germany (Fig. 5). We separated grasslands from other agricultural land use types because they provide a specifically bee-friendly and usually large habitat in the agricultural landscape and were most frequently sampled (97 database entries). The category ‘agriculture’ also included fields and small-scale edge structures, such as hedges and flower strips. Urban areas, including parks and botanical gardens, were among the most frequently sampled land use types, contributing the second largest number of bee records in our database (77 entries), after grasslands. There was also a large number of records from studies that sampled specific bee-friendly habitats, such as ‘pits’, including former quarry pits, mainly from North Rhine-Westphalia, or ‘dams’, including dunes and dams. Forest samples in our database were typically taken at forest edges or on meadows and forest clearings within a larger forest matrix.

**Fig. 6:**
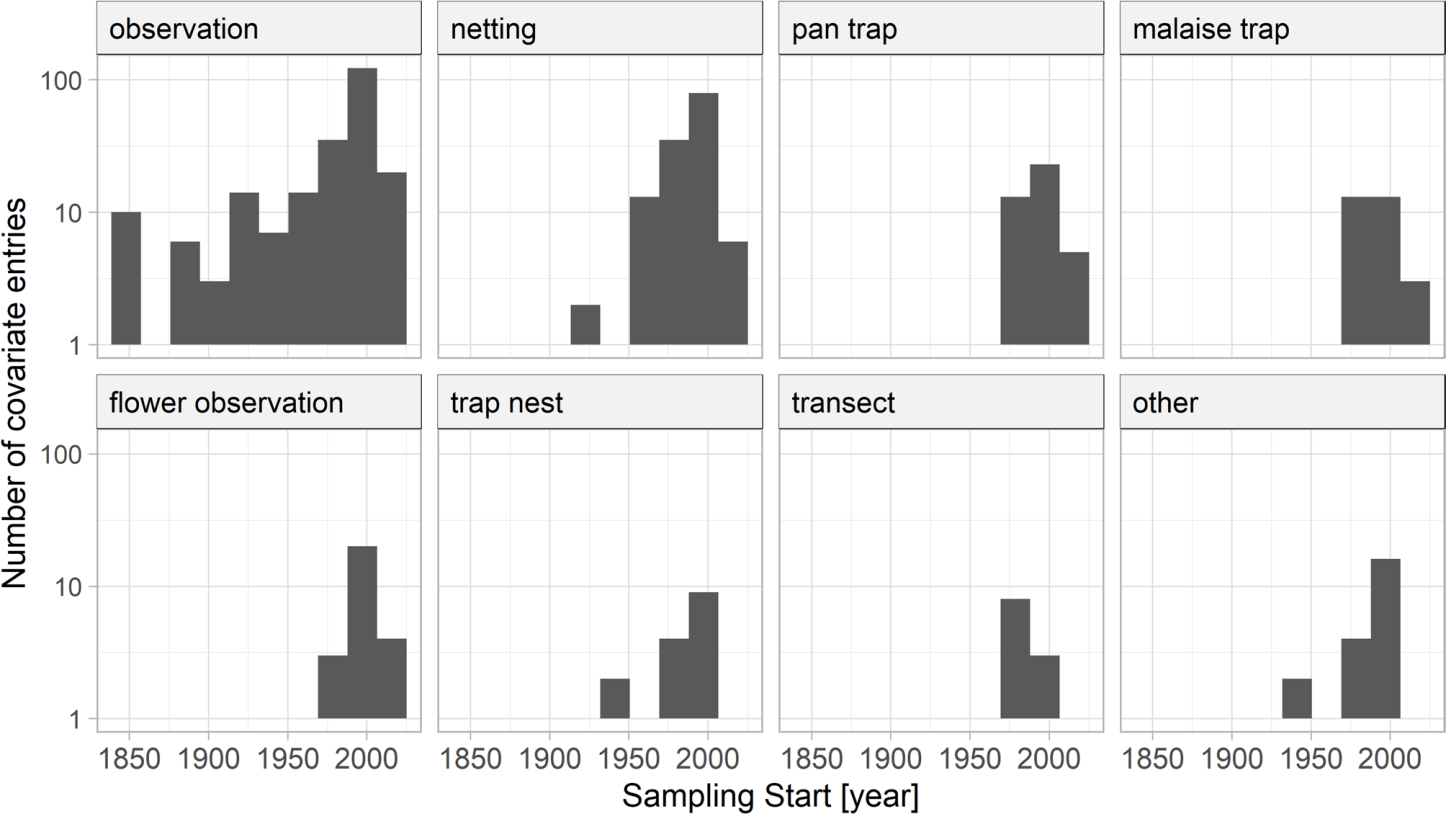
Bee sampling methods used. The category ‘other’ reflects entries with few records, such as emergence traps

**Fig. 7:**
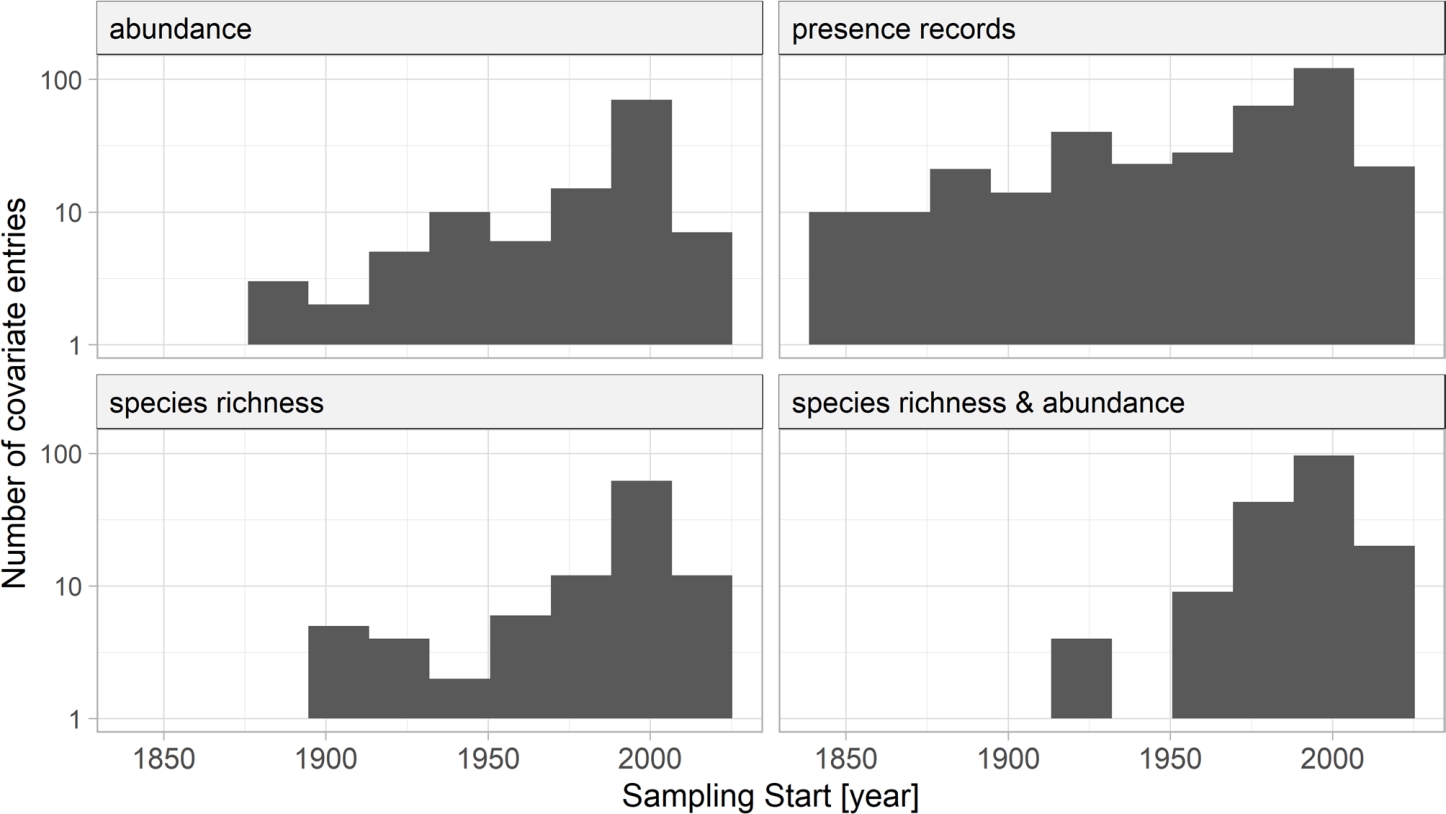
Wild bee records are given as abundance, presence records, species richness, or species richness and abundance

Bee sampling was carried out with different sampling methods, such as aerial netting or pitfall trapping. Bee-sampling methods were grouped into eight categories. Most records were from observations (227 entries, Fig. 6). Observations were either systematic, with detailed information on sampling effort (time spent searching for bees or area covered), or unsystematic. Older records, in particular, presented approaches such as intensive sampling of an area without specifying the size of the area or the time spent searching for bees. Therefore, most of the older records were presence-only data, reported as presence records without sampling effort or additional abundance information, as illustrated in Fig. 7. Such data are difficult to use in statistical analyses, but provide the best knowledge from bee records prior to 1950. Systematic netting, including aerial and sweep netting along transects, was the second most common method with 139 recorded entries. Similar to other more recently developed techniques, such as Malaise traps, trap nests, and pan traps, systematic netting was mainly used in studies starting around 1950. The category ‘other’ includes sampling methods with less than 10 entries in the database, such as pitfall traps, emergence traps, or suction sampling.

At best, the recorded data can be used to estimate species richness and abundance at specific locations and times. The ‘wildbee records’ column in the database provides an overview of the type of data available from each study, such as presence-only records or information on species richness and abundance (Fig. 7). Most studies provided only presence records, which do not allow conclusions on abundance or comparisons between years within the same data source. The type of data was closely linked to the sampling method. For example, observations often resulted in presence records but occasionally included measures of abundance and species richness. Conversely, Malaise traps rarely provided presence-only records and predominantly yielded abundance and species richness data. Presence records were by far the most common type of data reported and had the longest historical coverage. However, some studies from as early as the 1900s also included abundance and species richness data (Fig. 7).

**Fig. 8:**
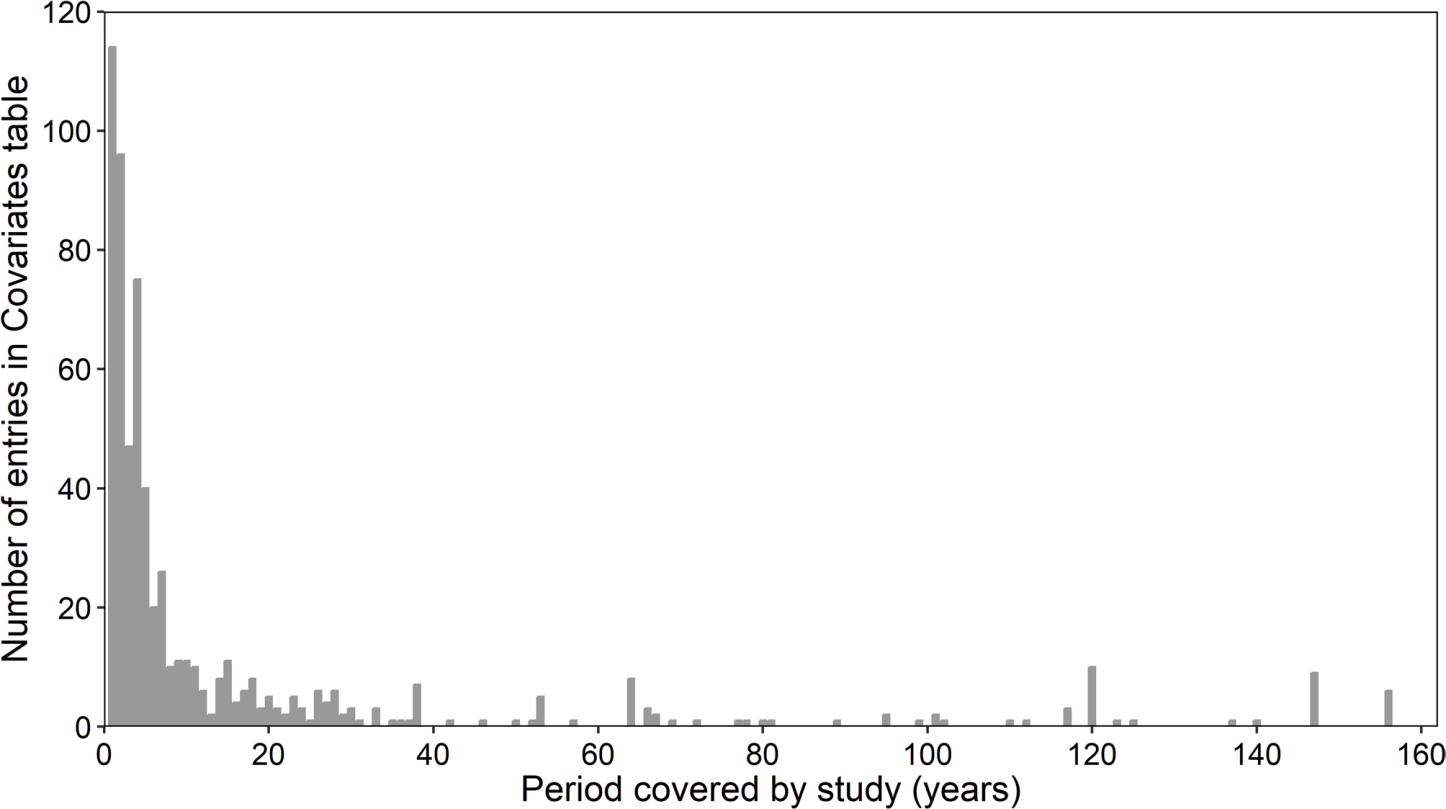
Number of entries in the Covariate Table per number of studies covered (in years). The period covered equals the difference between the start and end year

The study duration was often short, covering only a few years and only in exceptional cases more than 20 years (Fig. 8). Most studies with longer study durations did not cover the entire study period with regular annual sampling protocols, but rather samples with long sampling intervals in between. The average of all studies covered a sampling period of fours years (median), while one quarter of all studies covered only the minimum number of two years (first interquartile). That highlights that most studies covered only a short time span.

### Wild bee diversity in two selected German regions (Hesse and Saxony-Anhalt) over time (Data Table)

**Fig. 9:**
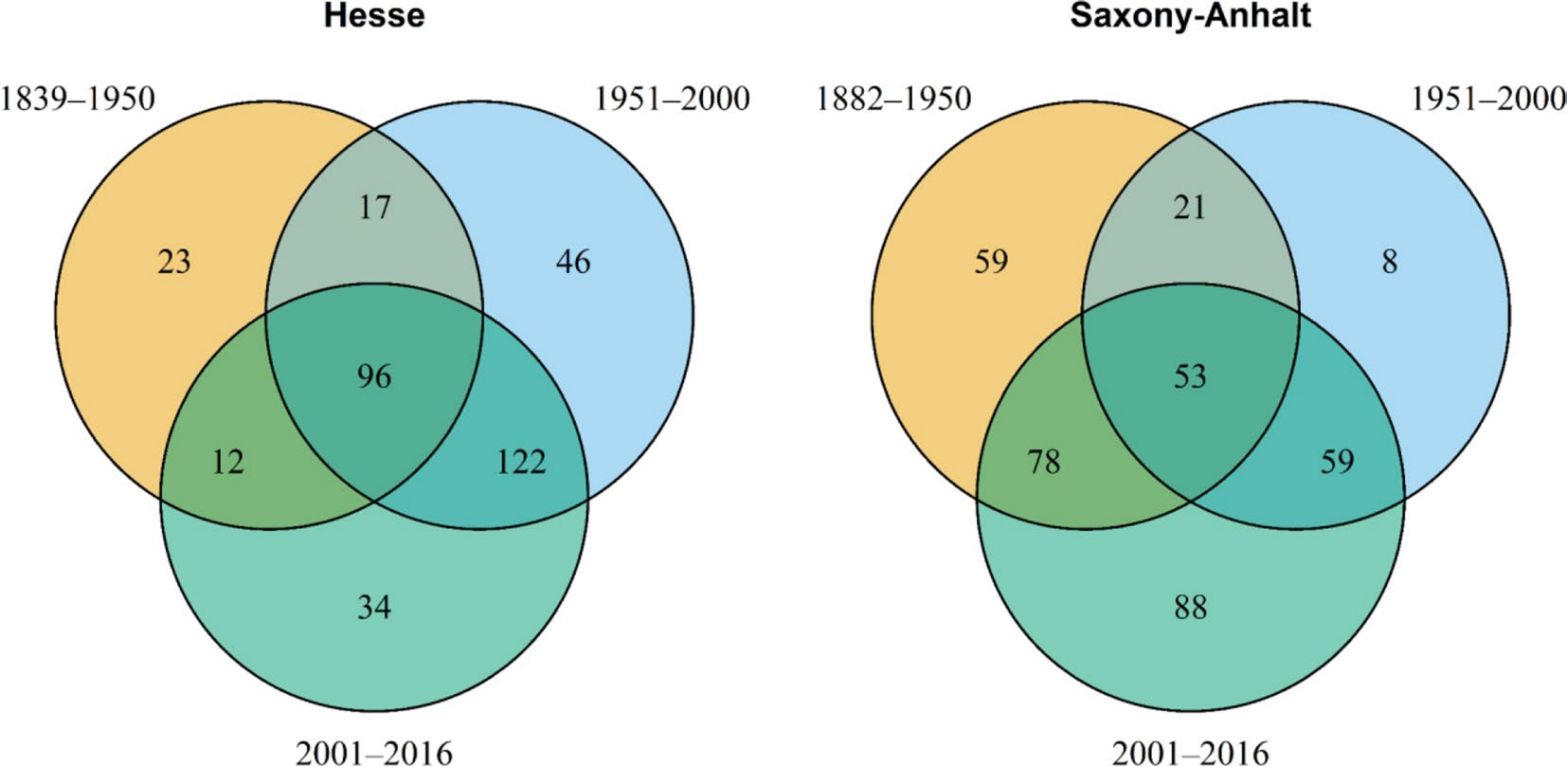
Venn diagrams illustrating the numbers of wild bee species in Hesse (left) and Saxony-Anhalt (right) over three different time periods

The Data Table will provide detailed information on the data from every study. Each sampling event will have an entry specifying the recorded bee species, the date, and the location. This will allow an analysis of bee trends from the start of sampling in the late 19th century to the present. To illustrate the potential of the database and particularly the Data Table, we extracted wild bee records from the federal states Hesse and Saxony-Anhalt and conducted an exemplary descriptive analysis. The table includes 27,436 data records in total, of which 23,578 records originate from the TERENO wild bee monitoring dataset of Saxony-Anhalt [52]. Our descriptive analysis (Fig. 9) highlights the number of bee species recorded in each time period (additional file S8). In both regions, the overlap of bee species sampled across all time periods is relatively low compared to the number of species found exclusively in one of the three periods. This result suggests potential changes and trends in bee communities over time.

### Limitations of the map

We have introduced a database containing studies on wild bee trends in Germany. The database consists of three tables, the first containing study-specific information, such as authors, title, year of publication, and journal. The search covered databases with peer-reviewed literature as well as German-language journals and inquiries with experts and state authorities. A key limitation of this search approach is its time intensity, as it requires a prolonged effort and the involvement of multiple researchers. Consequently, the resulting systematic map was conducted in November 2020 and is thus not fully up to date at the time of the publication due to the extended duration of the search and data extraction process. On the other hand, a major strength lies in the comprehensive temporal coverage: the data span as far back as 1885. The inclusion and digitization of pre-internet records provide a valuable foundation for future analyses.

The primary limitation of the database is the change in sampling methods over time, which restricts the comparability of data across different periods. Earlier records were often based on natural history-driven, opportunistic observations, whereas more recent data are typically generated using standardized methods such as transect walks, Malaise traps, or pan traps. In the coming years, a further shift is expected from specimen sampling and manual species identification by taxonomists to (semi-)automated process. This will allow the processing of large amounts of data, such as DNA metabarcoding, eDNA sampling, or automated visual and audio recording with computational visual analysis via artificial intelligence and large training datasets [53, 54]. For trend analysis, the challenge is to combine data sampled by different methods (which change continuously over time), with different spatio-temporal resolution and repeatability, and potential variations in data quality. One promising development, among others, is the increasing use of occupancy models in Europe and beyond [55–59].

## Conclusion

### Implication for policy

Our database contains bee data from all over Germany from 1885 up to November 2020, sampled over at least two years at the same location and with the same sampling protocol. Since the beginning of the 21st century, research on wild bee trends has further shifted towards more systematic inventories in line with an increased awareness of pollinator decline and streamlined concepts like ecosystem services, which highlight the importance of pollination for human well-being [13, 22, 24]. More recently, increasing evidence of pollinator declines and new requirements from environmental legislation have stimulated research into standardized large-scale monitoring of wild bee communities and other pollinators [60, 61]. Thus, published research on bee trends has generally followed the increasing academic standards in entomological survey design and methodology. If Germany and Europe as a whole are to make a political commitment to the protection and conservation of bees and other pollinators, it is important to have reliable data and information on insects and their local and global changes.

### Implication for research

The database can be used for future analyses of wild bee trends and for assessing the drivers of these trends and the potential success of mitigation measures. Syntheses aiming to analyse trends, drivers for trends, and mitigation measures have to take into account that primary data have been assembled with various different sampling approaches. Thus, particular methods are required to consider the different nature of data and a small data size given the low number of replicates, e.g., for some land use types.

## Electronic supplementary material

Below is the link to the electronic supplementary material.

Supplementary Material 1: Additional file S1. Search term for searches in Web of Science and Scopus. Additional file S2. Search record. Additional file S3. ROSES form for systematic maps. Additional file S4. R code and data. Additional file S5. Excluded full text records with reasons for exclusion. Additional file S6. Unretrievable full texts. Additional file S7. Study clusters. Additional file S8. Species List of Hesse and Saxony-Anhalt, German.

## Data Availability

Data is provided as supplementary information files.
