# Supplementary material for: What evidence exists on wild bee trends in Germany? A systematic map
Source: Environ Evid. 2025 Jun 19;14:11. doi: 10.1186/s13750-025-00364-7 (PMC12178071; doi:10.1186/s13750-025-00364-7)
Supplement: Supplementary file 1 — Supplementary Material 1: Additional file S1. Search term for searches in Web of Science and Scopus. Additional file S2. Search record. Additional file S3. ROSES form for systematic maps. Additional file S4. R code and data. Additional file S5. Excluded full text records with reasons for exclusion. Additional file S6. Unretrievable full texts. Additional file S7. Study clusters. Additional file S8. Species List of Hesse and Saxony-Anhalt, German. [file 13750_2025_364_MOESM1_ESM.zip › Supplements Environ Evid (2025-05-15)/Mupepele_Hellwig_et_al_2025_S1_Search_strings.pdf]

Mupepele, Hellwig, et al. (2025), Supplement S1, Search strings

Web of Science

(TS=(German* OR Deutsch* OR "Baden-Wuerttemberg" OR "Baden-Württemberg$" OR Bavaria OR Bayern$ OR Berlin$ OR Brandenburg$ OR Bremen$ OR Hamburg$ OR Hesse OR Hessen$ OR "Mecklenburg-Western Pomerania" OR "Mecklenburg-Vorpommern$" OR "Lower Saxony" OR Niedersachsen$ OR "North Rhine-Westphalia" OR "Nordrhein-Westfalen$" OR "Rhineland-Palatinate" OR "Rheinland-Pfalz" OR Saarland* OR Saxony OR Sachsen$ OR "Saxony-Anhalt" OR "Sachsen-Anhalt$" OR "Schleswig-Holstein$" OR Thuringia OR Thüringen$) OR AD=(Germany OR Deutschland OR "Baden-Wuerttemberg" OR "Baden-Württemberg" OR Bavaria OR Bayern OR Berlin OR Brandenburg OR Bremen OR Hamburg OR Hesse OR Hessen OR "Mecklenburg-Western Pomerania" OR "Mecklenburg-Vorpommern" OR "Lower Saxony" OR Niedersachsen OR "North Rhine-Westphalia" OR "Nordrhein-Westfalen" OR "Rhineland-Palatinate" OR "Rheinland-Pfalz" OR Saarland OR Saxony OR Sachsen OR "Saxony-Anhalt" OR "Sachsen-Anhalt" OR "Schleswig-Holstein" OR Thuringia OR Thüringen)) AND TS=("wild bee$" OR "trap nesting bee$" OR “cavity nesting bee$” OR "ground nesting bee$" OR "solitary bee$" OR Anthophila OR Apiformes OR Apoidea OR Andrenid* OR Apid* OR Colletid* OR Halictid* OR Megachilid* OR Melittid* OR Ammobates OR Ammobatoides OR Andrena OR Anthidium OR Anthophora OR Biastes OR Bombus OR Camptopoeum OR Ceratina OR Chelostoma OR Coelioxys OR Colletes OR Dasypoda OR Dioxys OR Dufourea OR Epeoloides OR Epeolus OR Eucera OR Halictus OR Heriades OR Hylaeus OR Lasioglossum OR Lithurgus OR Macropis OR Megachile OR Melecta OR Melitta OR Melitturga OR Nomada OR Nomia OR Nomioides OR Osmia OR Panurginus OR Panurgus OR Rhophitoides OR Rophites OR Sphecodes OR Stelis OR Systropha OR Thyreus OR Xylocopa OR "mining bee$" OR "carder bee$" OR "potter bee$" OR "flower bee$" OR "digger bee$" OR bumblebee$ OR "bumble bee$" OR "carpenter bee$" OR "scissor bee$" OR "sharp-tail bee$" OR "plasterer bee$" OR "vernal bee$" OR "ivy bee$" OR "sea aster bee$" OR "hairy-legged bee$" OR "pantaloon bee$" OR "long-horned bee$" OR "furrow bee$" OR "resin bee$" OR "yellow-faced bee$" OR "yellow-face bee$" OR "yellow loosestrife bee$" OR "leafcutter bee$" OR "leaf-cutter bee$" OR "leafcutting bee$" OR "leaf-cutting bee$" OR "sainfoin bee$" OR "red bartisia bee$" OR "nomad bee$" OR "mason bee$" OR "shaggy bee$" OR "gray-haired bee$" OR "alfalfa bee$" OR "blood bee$" OR "dark bee$" OR "spiral-horned bee$" OR "cuckoo bee$" OR pollinat* OR Wildbiene$ OR Sandgängerbiene$ OR Steppenglanzbiene$ OR Sandbiene$ OR Wollbiene$ OR Harzbiene$ OR Pelzbiene$ OR Kraftbiene$ OR Hummel$ OR Buntbiene$ OR Keulhornbiene$ OR Scherenbiene$ OR Kegelbiene$ OR Seidenbiene$ OR Hosenbiene$ OR Zweizahnbiene$ OR Glanzbiene$ OR Schmuckbiene$ OR Filzbiene$ OR Langhornbiene$ OR Furchenbiene$ OR Löcherbiene$ OR Maskenbiene$ OR Schmalbiene$ OR Steinbiene$ OR Schenkelbiene$ OR Blattschneiderbiene$ OR Mörtelbiene$ OR Trauerbiene$ OR Sägehornbiene$ OR Schwebebiene$ OR Wespenbiene$ OR Schienenbiene$ OR Steppenbiene$ OR Mauerbiene$ OR Scheinlappenbiene$ OR Zottelbiene$ OR Graubiene$ OR Schlürfbiene$ OR Buckelbiene$ OR Düsterbiene$ OR Spiralhornbiene$ OR Fleckenbiene$ OR Holzbiene$ OR Bestäub*)

Scopus

(TITLE-ABS-KEY(German* OR Deutsch* OR "Baden-Wuerttemberg" OR "Baden-Württemberg*" OR Bavaria OR Bayern* OR Berlin* OR Brandenburg* OR Bremen* OR Hamburg* OR Hesse OR Hessen* OR "Mecklenburg-Western Pomerania" OR "Mecklenburg-Vorpommern*" OR "Lower Saxony" OR Niedersachsen* OR "North Rhine-Westphalia" OR "Nordrhein-Westfalen*" OR "Rhineland-Palatinate" OR "Rheinland-Pfalz" OR Saarland* OR Saxony OR Sachsen* OR "Saxony-Anhalt" OR "Sachsen-Anhalt*" OR "Schleswig-Holstein*" OR Thuringia OR Thüringen*) OR AFFIL(Germany OR Deutschland OR "Baden-Wuerttemberg" OR "Baden-Württemberg" OR Bavaria OR Bayern OR Berlin OR Brandenburg OR Bremen OR Hamburg OR Hesse OR Hessen OR "Mecklenburg-Western Pomerania" OR "Mecklenburg-Vorpommern" OR "Lower Saxony" OR Niedersachsen OR "North Rhine-Westphalia" OR "Nordrhein-Westfalen" OR "Rhineland-Palatinate" OR "Rheinland-Pfalz" OR Saarland OR Saxony OR Sachsen OR "Saxony-Anhalt" OR "Sachsen-Anhalt" OR "Schleswig-Holstein" OR Thuringia OR Thüringen))AND TITLE-ABS-KEY("wild bee*" OR "trap nesting bee*" OR “cavity nesting bee*” OR "ground nesting bee*" OR "solitary bee*" OR Anthophila OR Apiformes OR Apoidea OR Andrenid* OR Apid* OR Colletid* OR Halictid* OR Megachilid* OR Melittid* OR Ammobates OR Ammobatoides OR Andrena OR Anthidium OR Anthophora OR Biastes OR Bombus OR Camptopoeum OR Ceratina OR Chelostoma OR Coelioxys OR Colletes OR Dasypoda OR Dioxys OR Dufourea OR Epeoloides OR Epeolus OR Eucera OR Halictus OR Heriades OR Hylaeus OR Lasioglossum OR Lithurgus OR Macropis OR Megachile OR Melecta OR Melitta OR Melitturga OR Nomada OR Nomia OR Nomioides OR Osmia OR Panurginus OR Panurgus OR Rhophitoides OR Rophites OR Sphecodes OR Stelis OR Systropha OR Thyreus OR Xylocopa OR "mining bee*" OR "carder bee*" OR "potter bee*" OR "flower bee*" OR "digger bee*" OR bumblebee* OR "bumble bee*" OR "carpenter bee*" OR "scissor bee*" OR "sharp-tail bee*" OR "plasterer bee*" OR "vernal bee*" OR "ivy bee*" OR "sea aster bee*" OR "hairy-legged bee*" OR "pantaloon bee*" OR "long-horned bee*" OR "furrow bee*" OR "resin bee*" OR "yellow-faced bee*" OR "yellow-face bee*" OR "yellow loosestrife bee*" OR "leafcutter bee*" OR "leaf-cutter bee*" OR "leafcutting bee*" OR "leaf-cutting bee*" OR "sainfoin bee*" OR "red bartisia bee*" OR "nomad bee*" OR "mason bee*" OR "shaggy bee*" OR "gray-haired bee*" OR "alfalfa bee*" OR "blood bee*" OR "dark bee*" OR "spiral-horned bee*" OR "cuckoo bee*" OR pollinat* OR Wildbiene* OR Sandgängerbiene* OR Steppenglanzbiene* OR Sandbiene* OR Wollbiene* OR Harzbiene* OR Pelzbiene* OR Kraftbiene* OR Hummel* OR Buntbiene* OR Keulhornbiene* OR Scherenbiene* OR Kegelbiene* OR Seidenbiene* OR Hosenbiene* OR Zweizahnbiene* OR Glanzbiene* OR Schmuckbiene* OR Filzbiene* OR Langhornbiene* OR Furchenbiene* OR Löcherbiene* OR Maskenbiene* OR Schmalbiene* OR Steinbiene* OR Schenkelbiene* OR Blattschneiderbiene* OR Mörtelbiene* OR Trauerbiene* OR Sägehornbiene* OR Schwebebiene* OR Wespenbiene* OR Schienenbiene* OR Steppenbiene* OR Mauerbiene* OR Scheinlappenbiene* OR Zottelbiene* OR Graubiene* OR Schlürfbiene* OR Buckelbiene* OR Düsterbiene* OR Spiralhornbiene* OR Fleckenbiene* OR Holzbiene* OR Bestäub*)
