# Supplementary material for: What evidence exists on wild bee trends in Germany? A systematic map
Source: Environ Evid. 2025 Jun 19;14:11. doi: 10.1186/s13750-025-00364-7 (PMC12178071; doi:10.1186/s13750-025-00364-7)
Supplement: Supplementary file 1 — Supplementary Material 1: Additional file S1. Search term for searches in Web of Science and Scopus. Additional file S2. Search record. Additional file S3. ROSES form for systematic maps. Additional file S4. R code and data. Additional file S5. Excluded full text records with reasons for exclusion. Additional file S6. Unretrievable full texts. Additional file S7. Study clusters. Additional file S8. Species List of Hesse and Saxony-Anhalt, German. [file 13750_2025_364_MOESM1_ESM.zip › Supplements Environ Evid (2025-05-15)/Mupepele_Hellwig_et_al_2025_S8_Species_list.pdf]

Mupepele, Hellwig, et al. (2025), Supplement S8, Species List of Hesse

	prior to 1950	1950 to 2000	after 2000
Ammobates punctatus	0	0	1
Andrena agilissima	0	0	1
Andrena alfkenella	0	1	0
Andrena angustior	0	1	1
Andrena argentata	0	0	1
Andrena barbilabris	0	1	1
Andrena bicolor	1	1	1
Andrena bimaculata	0	1	1
Andrena chrysopus	0	1	1
Andrena chrysopyga	1	1	0
Andrena chrysosceles	0	1	1
Andrena cineraria	1	1	1
Andrena clarkella	1	1	1
Andrena coitana	0	1	0
Andrena combinata	1	1	1
Andrena congruens	1	0	0
Andrena curvungula	1	1	1
Andrena decipiens	1	1	0
Andrena denticulata	1	1	0
Andrena dorsata	1	1	1
Andrena falsifica	0	0	1
Andrena ferox	1	1	0
Andrena flavilabris	0	1	0
Andrena flavipes	1	1	1
Andrena florea	0	1	0
Andrena floricola	0	1	0
Andrena fucata	0	1	1
Andrena fulva	1	1	1
Andrena fulvago	1	1	1
Andrena fulvata	0	0	1
Andrena fulvida	0	1	0
Andrena fuscipes	1	1	1
Andrena gelriae	0	1	1
Andrena gravida	0	1	1
Andrena haemorrhoa	1	1	1
Andrena hattorfiana	1	1	1
Andrena helvola	1	1	1
Andrena humilis	1	1	1
Andrena intermedia	0	1	0
Andrena labialis	0	1	1
Andrena labiata	1	1	1
Andrena lagopus	0	1	1
Andrena lathyri	0	1	1
Andrena lepida	0	1	0
Andrena minutula	1	1	1
Andrena minutuloides	0	1	1
Andrena mitis	0	1	1
Andrena nana	1	0	0
Andrena nigroaenea	1	1	1

	prior to 1950	1950 to 2000	after 2000
Andrena nitida	1	1	1
Andrena nitidiuscula	0	0	1
Andrena nuptialis	0	0	1
Andrena ovatula	0	1	1
Andrena pandellei	0	1	1
Andrena pilipes	1	0	1
Andrena potentillae	1	1	1
Andrena praecox	1	1	1
Andrena proxima	0	1	1
Andrena rosae	1	0	0
Andrena rugulosa	0	0	1
Andrena scotica	0	1	1
Andrena semilaevis	0	1	0
Andrena similis	0	1	0
Andrena strohmella	0	1	1
Andrena subopaca	0	1	1
Andrena synadelpha	0	1	0
Andrena thoracica	1	0	0
Andrena tibialis	1	1	1
Andrena trimmerana	1	0	0
Andrena tscheki	1	0	0
Andrena vaga	1	1	1
Andrena varians	0	1	1
Andrena viridescens	1	1	1
Andrena wilkella	1	1	1
Anthidium byssinum	1	1	1
Anthidium manicatum	1	1	1
Anthidium nanum	1	1	1
Anthidium oblongatum	0	1	1
Anthidium punctatum	0	1	1
Anthidium strigatum	1	1	1
Anthophora aestivalis	0	1	1
Anthophora bimaculata	1	0	1
Anthophora furcata	1	1	1
Anthophora plumipes	0	1	1
Anthophora quadrifasciata	1	0	0
Anthophora quadrimaculata	1	0	1
Anthophora retusa	1	1	1
Biastes truncatus	1	1	1
Bombus barbutellus	1	1	1
Bombus bohemicus	0	1	1
Bombus campestris	1	1	1
Bombus confusus	1	1	0
Bombus cryptarum	0	0	1
Bombus distinguendus	1	1	0
Bombus hortorum	1	1	1
Bombus humilis	0	1	1
Bombus hypnorum	1	1	1
Bombus jonellus	0	1	0

	prior to 1950	1950 to 2000	after 2000
Bombus lapidarius	1	1	1
Bombus lucorum	1	1	1
Bombus magnus	0	1	0
Bombus muscorum	1	1	0
Bombus norvegicus	1	1	1
Bombus pascuorum	1	1	1
Bombus pomorum	1	1	0
Bombus pratorum	1	1	1
Bombus quadricolor	1	0	0
Bombus ruderarius	1	1	1
Bombus ruderatus	1	1	0
Bombus rupestris	1	1	1
Bombus soroeensis	1	1	1
Bombus subterraneus	1	1	0
Bombus sylvarum	1	1	1
Bombus sylvestris	0	1	1
Bombus terrestris	1	1	1
Bombus variabilis	1	0	0
Bombus vestalis	1	1	1
Bombus veteranus	1	1	1
Bombus wurflenii	1	1	0
Ceratina chalybea	0	1	1
Ceratina cucurbitina	0	1	1
Ceratina cyanea	0	1	1
Chelostoma distinctum	0	1	1
Coelioxys afra	0	1	1
Coelioxys aurolimbata	0	1	0
Coelioxys conica	1	0	1
Coelioxys conoidea	0	1	1
Coelioxys elongata	0	0	1
Coelioxys inermis	1	1	1
Coelioxys mandibularis	0	0	1
Coelioxys rufescens	1	0	0
Colletes cunicularius	0	1	1
Colletes daviesanus	1	1	1
Colletes fodiens	0	0	1
Colletes hederae	0	0	1
Colletes similis	0	1	0
Colletes succinctus	0	0	1
Dasypoda hirtipes	1	0	1
Dufourea dentiventris	1	1	1
Dufourea halictula	1	1	0
Dufourea inermis	1	1	0
Dufourea minuta	1	1	1
Epeoloides coecutiens	0	1	1
Epeolus cruciger	0	0	1
Epeolus variegatus	1	1	1
Eucera longicornis	1	1	1
Eucera macroglossa	1	0	1

	prior to 1950	1950 to 2000	after 2000
Eucera nigrescens	0	1	1
Halictus confusus	1	0	0
Halictus eurygnathus	0	1	0
Halictus langobardicus	0	0	1
Halictus leucaheneus	0	0	1
Halictus ligatus	1	0	0
Halictus maculatus	1	1	1
Halictus quadricinctus	1	0	0
Halictus rubicundus	1	1	1
Halictus scabiosae	1	1	1
Halictus sexcinctus	1	0	1
Halictus simplex	0	1	1
Halictus smaragdulus	0	0	1
Halictus subauratus	0	0	1
Halictus tetrazonius	1	0	0
Halictus tumulorum	0	1	1
Hylaeus angustatus	1	1	1
Hylaeus annularis	0	1	1
Hylaeus brevicornis	0	1	1
Hylaeus clypearis	0	1	1
Hylaeus communis	0	1	1
Hylaeus confusus	1	1	1
Hylaeus cornutus	0	1	0
Hylaeus difformis	0	1	1
Hylaeus duckei	0	1	1
Hylaeus gibbus	0	1	1
Hylaeus gracilicornis	0	1	0
Hylaeus gredleri	0	1	1
Hylaeus hyalinatus	0	1	1
Hylaeus incongruus	0	1	1
Hylaeus leptocephalus	0	1	0
Hylaeus lineolatus	0	0	1
Hylaeus moricei	0	1	0
Hylaeus nigritus	0	1	1
Hylaeus paulus	0	1	1
Hylaeus pictipes	0	1	1
Hylaeus punctatus	0	1	0
Hylaeus punctulatissimus	0	1	1
Hylaeus rinki	0	1	0
Hylaeus signatus	0	0	1
Hylaeus sinuatus	0	1	1
Hylaeus styriacus	0	1	1
Hylaeus variegatus	1	1	1
Lasioglossum albipes	0	1	1
Lasioglossum angusticeps	0	1	0
Lasioglossum brevicorne	0	0	1
Lasioglossum calceatum	1	1	1
Lasioglossum costulatum	0	1	1
Lasioglossum fratellum	0	1	1

	prior to 1950	1950 to 2000	after 2000
Lasioglossum fulvicorne	0	1	1
Lasioglossum glabriusculum	0	1	0
Lasioglossum intermedium	0	1	0
Lasioglossum interruptum	0	0	1
Lasioglossum laevigatum	0	1	1
Lasioglossum laticeps	0	1	1
Lasioglossum lativentre	0	1	1
Lasioglossum leucopus	0	1	1
Lasioglossum leucozonium	1	1	1
Lasioglossum lucidulum	0	0	1
Lasioglossum malachurum	0	1	1
Lasioglossum minutissimum	0	1	1
Lasioglossum minutulum	0	1	1
Lasioglossum morio	1	1	1
Lasioglossum nitidiusculum	1	1	1
Lasioglossum nitidulum	0	1	1
Lasioglossum parvulum	0	1	1
Lasioglossum pauperatum	0	1	0
Lasioglossum pauxillum	0	1	1
Lasioglossum politum	0	1	1
Lasioglossum punctatissimum	0	1	1
Lasioglossum pygmaeum	0	0	1
Lasioglossum rufitarse	0	1	1
Lasioglossum semilucens	0	1	1
Lasioglossum sexnotatum	1	0	1
Lasioglossum sexstrigatum	0	0	1
Lasioglossum smeathmanellum	0	1	1
Lasioglossum subfasciatum	1	0	0
Lasioglossum villosulum	1	1	1
Lasioglossum xanthopus	0	1	0
Lasioglossum zonulum	1	1	1
Macropis europaea	0	1	1
Macropis fulvipes	0	1	1
Megachile alpicola	0	1	0
Megachile centuncularis	1	1	1
Megachile circumcincta	0	1	1
Megachile ericetorum	0	1	1
Megachile lagopoda	1	1	1
Megachile lapponica	0	1	1
Megachile ligniseca	1	1	1
Megachile maritima	1	1	0
Megachile nigriventris	0	1	1
Megachile pilidens	0	1	1
Megachile rotundata	0	1	0
Megachile versicolor	0	1	1
Megachile willughbiella	1	1	1
Melecta albifrons	0	1	1
Melecta luctuosa	1	1	0
Melitta haemorrhoidalis	1	1	1

	prior to 1950	1950 to 2000	after 2000
Melitta leporina	1	0	1
Melitta nigricans	0	1	1
Melitta tricincta	0	1	1
Melitturga clavicornis	1	0	0
Nomada alboguttata	1	0	1
Nomada armata	1	1	1
Nomada atroscutellaris	0	1	1
Nomada baccata	0	1	0
Nomada bifasciata	0	1	1
Nomada braunsiana	0	1	0
Nomada capriciana	0	1	0
Nomada castellana	0	1	1
Nomada conjungens	0	1	1
Nomada distinguenda	1	0	0
Nomada fabriciana	0	1	1
Nomada facilis	0	1	0
Nomada femoralis	1	1	1
Nomada ferruginata	1	1	1
Nomada flava	0	1	1
Nomada flavoguttata	1	1	1
Nomada flavopicta	1	1	1
Nomada fucata	1	1	1
Nomada fulvicornis	1	1	1
Nomada fuscicornis	0	0	1
Nomada goodeniana	0	1	1
Nomada guttulata	0	1	0
Nomada hirtipes	0	0	1
Nomada integra	0	1	0
Nomada lathburiana	1	1	1
Nomada leucophthalma	1	0	1
Nomada marshamella	1	1	1
Nomada panzeri	0	1	1
Nomada piccioliana	0	1	1
Nomada rhenana	1	0	0
Nomada roberjeotiana	1	0	0
Nomada ruficornis	1	1	1
Nomada rufipes	1	1	1
Nomada sexfasciata	0	1	1
Nomada sheppardana	0	1	1
Nomada signata	0	1	1
Nomada similis	0	1	1
Nomada stigma	0	0	1
Nomada striata	1	1	1
Nomada succincta	1	1	1
Nomada villosa	1	1	1
Osmia adunca	0	1	1
Osmia aurulenta	1	1	1
Osmia bicolor	1	1	1
Osmia bicornis	1	1	1

	prior to 1950	1950 to 2000	after 2000
Osmia brevicornis	0	0	1
Osmia caerulescens	1	1	1
Osmia campanularum	0	1	1
Osmia cantabrica	0	1	0
Osmia claviventris	0	1	0
Osmia cornuta	1	1	0
Osmia florisomnis	1	1	1
Osmia gallarum	0	1	1
Osmia inermis	0	1	0
Osmia leaiana	0	1	1
Osmia leucomelana	0	1	1
Osmia mitis	0	1	0
Osmia nigriventris	1	0	0
Osmia niveata	0	1	1
Osmia papaveris	1	0	0
Osmia parietina	0	1	1
Osmia pilicornis	1	0	0
Osmia rapunculi	1	1	1
Osmia ravouxi	0	1	1
Osmia rufohirta	0	0	1
Osmia spinulosa	0	1	1
Osmia tridentata	0	1	1
Osmia truncorum	0	1	1
Osmia uncinata	1	0	1
Panurgus banksianus	0	1	1
Panurgus calcaratus	1	1	1
Panurgus dentipes	0	1	1
Pseudapis femoralis	1	0	0
Rhophitoides canus	0	1	0
Sphecodes albilabris	1	1	0
Sphecodes crassus	0	1	1
Sphecodes croaticus	0	1	0
Sphecodes ephippius	1	1	1
Sphecodes ferruginatus	0	1	1
Sphecodes geoffrellus	0	1	1
Sphecodes gibbus	1	1	1
Sphecodes hyalinatus	0	1	1
Sphecodes longulus	0	1	1
Sphecodes marginatus	0	1	1
Sphecodes miniatus	0	1	0
Sphecodes monilicornis	1	1	1
Sphecodes niger	0	1	1
Sphecodes pellucidus	0	1	1
Sphecodes pseudofasciatus	0	0	1
Sphecodes puncticeps	0	1	1
Sphecodes reticulatus	0	0	1
Sphecodes rubicundus	0	1	0
Sphecodes rufiventris	0	1	1
Sphecodes scabricollis	0	1	0

	prior to 1950	1950 to 2000	after 2000
Sphecodes spinulosus	0	1	0
Stelis breviuscula	0	1	1
Stelis minuta	0	1	0
Stelis phaeoptera	0	0	1
Stelis punctulatissima	1	1	1
Thyreus orbatus	0	1	1
Xylocopa violacea	1	1	1

	prior to 1950	1950 to 2000	after 2000
Ammobates punctatus	1	1	0
Andrena agilissima	1	0	1
Andrena alfkenella	1	1	1
Andrena angustior	0	0	1
Andrena anthrisci	1	1	1
Andrena apicata	1	0	0
Andrena argentata	1	0	0
Andrena barbareae	1	0	0
Andrena barbilabris	1	1	1
Andrena bicolor	0	1	1
Andrena bimaculata	1	0	1
Andrena chrysopus	1	0	0
Andrena chrysopyga	1	0	0
Andrena chrysosceles	0	1	1
Andrena cineraria	1	0	1
Andrena clarkella	1	0	1
Andrena coitana	1	0	0
Andrena combinata	1	0	1
Andrena congruens	1	0	0
Andrena curvungula	1	0	1
Andrena denticulata	1	0	1
Andrena distinguenda	1	1	1
Andrena dorsata	1	1	1
Andrena enslinella	0	0	1
Andrena falsifica	1	0	1
Andrena ferox	1	0	1
Andrena flavipes	0	1	1
Andrena florea	1	1	1
Andrena floricola	1	0	1
Andrena florivaga	0	0	1
Andrena fucata	0	0	1
Andrena fulva	0	1	1
Andrena fulvago	0	1	1
Andrena fulvicornis	0	0	1
Andrena fuscipes	0	0	1
Andrena gelriae	0	0	1
Andrena granulosa	1	0	0
Andrena gravida	0	1	1
Andrena haemorrhoa	0	1	1
Andrena hattorfiana	0	1	0
Andrena helvola	0	1	1
Andrena humilis	1	1	1
Andrena hypopolia	1	0	1
Andrena intermedia	0	0	1
Andrena labialis	0	0	1
Andrena labiata	0	0	1
Andrena lapponica	0	0	1
Andrena lathyri	1	0	1
Andrena lepida	1	0	0

	prior to 1950	1950 to 2000	after 2000
Andrena minutula	0	1	1
Andrena minutuloides	1	1	1
Andrena mitis	1	0	1
Andrena nana	1	0	1
Andrena nigriceps	1	0	0
Andrena nigroaenea	0	1	1
Andrena nigrospina	0	0	1
Andrena nitida	1	1	1
Andrena nitidiuscula	0	0	1
Andrena niveata	1	1	1
Andrena ovatula	0	0	1
Andrena pandellei	1	0	1
Andrena pilipes	1	1	1
Andrena polita	1	0	0
Andrena potentillae	1	1	0
Andrena praecox	0	1	1
Andrena proxima	0	0	1
Andrena pusilla	1	1	0
Andrena ruficrus	0	0	1
Andrena rufizona	1	0	0
Andrena schencki	1	0	0
Andrena scotica	0	1	1
Andrena semilaevis	0	1	1
Andrena similis	1	0	1
Andrena simillima	1	0	0
Andrena strohmella	1	1	1
Andrena subopaca	0	1	1
Andrena suerinensis	0	0	1
Andrena synadelpha	0	0	1
Andrena tibialis	0	1	1
Andrena trimmerana	0	0	1
Andrena vaga	0	0	1
Andrena varians	0	0	1
Andrena ventralis	1	0	1
Andrena viridescens	1	0	1
Andrena wilkella	0	0	1
Anthidium byssinum	1	0	1
Anthidium manicatum	1	1	1
Anthidium nanum	1	0	0
Anthidium oblongatum	1	0	0
Anthidium punctatum	1	0	1
Anthidium strigatum	1	0	1
Anthidium tenellum	0	1	1
Anthophora aestivalis	1	1	1
Anthophora bimaculata	1	1	0
Anthophora furcata	1	1	1
Anthophora plagiata	1	1	0
Anthophora plumipes	1	1	1
Anthophora quadrimaculata	1	1	0

	prior to 1950	1950 to 2000	after 2000
Anthophora retusa	0	1	1
Biastes emarginatus	1	0	1
Biastes truncatus	1	0	0
Bombus barbutellus	1	0	1
Bombus bohemicus	1	0	1
Bombus campestris	1	0	1
Bombus cryptarum	0	0	1
Bombus hortorum	0	1	1
Bombus hypnorum	0	1	1
Bombus jonellus	0	0	1
Bombus lapidarius	0	1	1
Bombus lucorum	0	0	1
Bombus muscorum	0	0	1
Bombus norvegicus	0	0	1
Bombus pascuorum	0	1	1
Bombus pratorum	0	1	1
Bombus quadricolor	1	1	1
Bombus ruderarius	0	1	1
Bombus ruderatus	0	0	1
Bombus rupestris	0	1	1
Bombus semenoviellus	0	0	1
Bombus soroeensis	0	0	1
Bombus sylvarum	0	1	1
Bombus sylvestris	1	0	1
Bombus terrestris	0	1	1
Bombus vestalis	1	0	1
Bombus veteranus	0	1	0
Camptopoeum frontale	0	1	1
Ceratina cyanea	1	1	1
Coelioxys afra	1	1	0
Coelioxys aurolimbata	1	0	0
Coelioxys brevis	0	1	0
Coelioxys conica	1	0	1
Coelioxys conoidea	1	0	0
Coelioxys echinata	1	0	0
Coelioxys elongata	0	0	1
Coelioxys inermis	0	1	1
Coelioxys mandibularis	1	0	1
Coelioxys rufescens	0	0	1
Colletes cunicularius	0	1	1
Colletes daviesanus	0	1	1
Colletes fodiens	0	1	1
Colletes similis	1	0	1
Colletes succinctus	1	0	1
Dasypoda argentata	1	0	0
Dasypoda hirtipes	1	1	1
Dioxys tridentata	1	0	0
Dufourea dentiventris	0	0	1
Dufourea inermis	1	0	0

	prior to 1950	1950 to 2000	after 2000
Epeoloides coecutiens	1	0	0
Epeolus cruciger	1	0	0
Eucera dentata	1	0	0
Eucera interrupta	1	0	0
Eucera longicornis	1	1	1
Eucera macroglossa	1	1	1
Eucera nigrescens	1	0	1
Halictus confusus	0	0	1
Halictus eurygnathus	0	0	1
Halictus langobardicus	0	0	1
Halictus leucaheneus	0	0	1
Halictus maculatus	0	1	1
Halictus quadricinctus	1	0	1
Halictus rubicundus	0	1	1
Halictus sajoi	1	0	0
Halictus scabiosae	0	0	1
Halictus sexcinctus	1	0	0
Halictus simplex	1	0	1
Halictus subauratus	1	1	1
Halictus tetrazonius	0	0	1
Halictus tumulorum	1	1	1
Hylaeus angustatus	1	1	1
Hylaeus annularis	1	1	1
Hylaeus brevicornis	0	1	1
Hylaeus clypearis	1	1	0
Hylaeus communis	1	1	1
Hylaeus confusus	0	1	1
Hylaeus difformis	1	0	1
Hylaeus dilatatus	0	0	1
Hylaeus gibbus	1	1	0
Hylaeus gredleri	0	0	1
Hylaeus hyalinatus	0	1	1
Hylaeus leptocephalus	1	1	1
Hylaeus nigritus	1	0	0
Hylaeus paulus	0	0	1
Hylaeus pectoralis	0	1	1
Hylaeus pictipes	1	1	1
Hylaeus punctatus	0	1	1
Hylaeus punctulatissimus	1	1	0
Hylaeus signatus	0	1	1
Hylaeus sinuatus	1	1	1
Hylaeus styriacus	1	1	1
Hylaeus variegatus	1	1	1
Lasioglossum aeratum	1	1	1
Lasioglossum albipes	0	0	1
Lasioglossum brevicorne	0	0	1
Lasioglossum breviventre	1	0	0
Lasioglossum calceatum	0	1	1
Lasioglossum clypeare	1	0	1

	prior to 1950	1950 to 2000	after 2000
Lasioglossum convexiusculum	1	0	1
Lasioglossum fratellum	0	0	1
Lasioglossum fulvicorne	0	0	1
Lasioglossum intermedium	1	1	1
Lasioglossum interruptum	1	0	1
Lasioglossum laeve	1	0	0
Lasioglossum laevigatum	1	0	1
Lasioglossum laticeps	1	1	1
Lasioglossum lativentre	0	1	1
Lasioglossum leucopus	1	1	1
Lasioglossum leucozonium	0	1	1
Lasioglossum limbellum	1	1	0
Lasioglossum lineare	1	0	1
Lasioglossum lissonotum	1	0	0
Lasioglossum lucidulum	1	1	1
Lasioglossum majus	1	0	1
Lasioglossum malachurum	0	1	1
Lasioglossum marginellum	1	0	0
Lasioglossum minutissimum	1	1	1
Lasioglossum minutulum	1	1	1
Lasioglossum morio	0	1	1
Lasioglossum nitidiusculum	0	1	1
Lasioglossum nitidulum	0	1	1
Lasioglossum pallens	1	0	1
Lasioglossum parvulum	0	1	1
Lasioglossum pauxillum	0	1	1
Lasioglossum politum	1	1	1
Lasioglossum punctatissimum	1	1	1
Lasioglossum puncticolle	1	0	0
Lasioglossum pygmaeum	1	0	1
Lasioglossum quadrinotatulum	0	0	1
Lasioglossum quadrinotatum	0	1	1
Lasioglossum quadrisignatum	1	0	0
Lasioglossum rufitarse	1	0	1
Lasioglossum semilucens	1	1	1
Lasioglossum sexnotatum	0	1	1
Lasioglossum sexstrigatum	0	1	1
Lasioglossum subfasciatum	1	0	1
Lasioglossum tricinctum	1	0	1
Lasioglossum villosulum	0	1	1
Lasioglossum xanthopus	1	0	1
Lasioglossum zonulum	1	1	1
Macropis europaea	1	1	1
Macropis fulvipes	1	1	0
Megachile alpicola	1	0	1
Megachile apicalis	0	1	0
Megachile centuncularis	1	1	1
Megachile circumcincta	1	0	1
Megachile ericetorum	1	1	0

	prior to 1950	1950 to 2000	after 2000
Megachile lagopoda	1	1	0
Megachile lapponica	0	0	1
Megachile ligniseca	1	1	1
Megachile maritima	1	0	1
Megachile parietina	1	0	0
Megachile pilidens	1	1	0
Megachile pyrenaea	0	0	1
Megachile rotundata	1	1	0
Megachile versicolor	1	0	1
Megachile willughbiella	1	1	0
Melecta albifrons	1	0	1
Melecta luctuosa	1	1	0
Melitta haemorrhoidalis	0	1	0
Melitta leporina	0	1	1
Melitta melanura	0	1	0
Melitta nigricans	1	0	0
Melitta tricincta	1	1	0
Nomada alboguttata	0	0	1
Nomada argentata	1	0	0
Nomada atroscutellaris	1	0	0
Nomada bifasciata	1	0	1
Nomada braunsiana	1	0	0
Nomada castellana	0	0	1
Nomada conjungens	1	0	1
Nomada distinguenda	1	0	1
Nomada fabriciana	0	0	1
Nomada ferruginata	1	0	1
Nomada flava	0	0	1
Nomada flavoguttata	0	0	1
Nomada flavopicta	0	0	1
Nomada fucata	0	1	1
Nomada fulvicornis	0	0	1
Nomada fuscicornis	0	0	1
Nomada goodeniana	0	0	1
Nomada guttulata	1	0	0
Nomada lathburiana	0	0	1
Nomada leucophthalma	0	0	1
Nomada marshamella	0	1	1
Nomada moeschleri	0	0	1
Nomada mutabilis	1	0	0
Nomada mutica	1	0	0
Nomada nobilis	1	1	0
Nomada panzeri	0	0	1
Nomada rhenana	1	0	0
Nomada roberjeotiana	1	0	1
Nomada ruficornis	0	0	1
Nomada rufipes	1	0	1
Nomada sexfasciata	0	1	0
Nomada sheppardana	1	0	1

	prior to 1950	1950 to 2000	after 2000
Nomada signata	0	0	1
***Nomada* spec.**	1	0	0
Nomada stigma	1	0	1
Nomada striata	0	0	1
Nomada succincta	0	0	1
Nomada villosa	0	0	1
Nomada zonata	1	0	1
Osmia adunca	1	1	1
Osmia andrenoides	1	0	0
Osmia anthocopoides	1	1	1
Osmia aurulenta	0	0	1
Osmia bicolor	0	0	1
Osmia bicornis	1	1	1
Osmia brevicornis	1	1	1
Osmia caerulescens	1	1	1
Osmia campanularum	0	1	1
Osmia cantabrica	1	0	1
Osmia claviventris	0	0	1
Osmia crenulata	0	0	1
Osmia florisomnis	0	1	1
Osmia leaiana	0	0	1
Osmia leucomelana	1	0	1
Osmia mitis	1	0	0
Osmia niveata	1	0	1
Osmia papaveris	1	0	0
Osmia parietina	0	0	1
Osmia pilicornis	1	0	0
Osmia rapunculi	0	1	1
Osmia ravouxi	1	0	1
Osmia rufohirta	1	0	0
Osmia spinulosa	1	1	1
Osmia tridentata	0	0	1
Osmia truncorum	1	1	1
Osmia uncinata	1	0	1
Osmia villosa	0	0	1
Osmia xanthomelana	1	0	0
Panurgus banksianus	1	1	1
Panurgus calcaratus	0	1	1
Rhophitoides canus	1	0	1
Rophites algirus	1	0	1
Rophites quinquespinosus	1	0	1
Sphecodes albilabris	0	0	1
Sphecodes crassus	0	0	1
Sphecodes cristatus	1	0	0
Sphecodes croaticus	0	1	0
Sphecodes ephippius	0	0	1
Sphecodes ferruginatus	1	0	1
Sphecodes geoffrellus	0	0	1
Sphecodes gibbus	0	0	1

	prior to 1950	1950 to 2000	after 2000
Sphecodes hyalinatus	1	0	1
Sphecodes longulus	1	0	1
Sphecodes majalis	0	0	1
Sphecodes marginatus	0	0	1
Sphecodes miniatus	0	0	1
Sphecodes monilicornis	0	0	1
Sphecodes niger	1	0	0
Sphecodes pellucidus	1	0	1
Sphecodes puncticeps	0	0	1
Sphecodes rubicundus	0	0	1
Sphecodes rufiventris	1	0	1
Sphecodes scabricollis	0	0	1
Sphecodes spinulosus	1	0	1
Sphecodes zangherii	0	0	1
Stelis breviuscula	1	0	1
Stelis minima	1	0	0
Stelis minuta	1	0	0
Stelis odontopyga	1	0	0
Stelis ornatula	1	0	1
Stelis punctulatissima	1	0	0
Systropha curvicornis	1	1	0
Thyreus orbatus	1	0	0
Xylocopa violacea	1	0	1
