# Supplementary material for: What evidence exists on wild bee trends in Germany? A systematic map
Source: Environ Evid. 2025 Jun 19;14:11. doi: 10.1186/s13750-025-00364-7 (PMC12178071; doi:10.1186/s13750-025-00364-7)
Supplement: Supplementary file 1 — Supplementary Material 1: Additional file S1. Search term for searches in Web of Science and Scopus. Additional file S2. Search record. Additional file S3. ROSES form for systematic maps. Additional file S4. R code and data. Additional file S5. Excluded full text records with reasons for exclusion. Additional file S6. Unretrievable full texts. Additional file S7. Study clusters. Additional file S8. Species List of Hesse and Saxony-Anhalt, German. [file 13750_2025_364_MOESM1_ESM.zip › Supplements Environ Evid (2025-05-15)/Mupepele_Hellwig_et_al_2025_S6_Unretrievable_full_texts.pdf]

Mupepele, Hellwig, et al. (2025), Supplement S6, Unretrievable full texts

Not accessible

Alfken, J.D. (1951): Die Bienenfauna von Bremen. Mitteilungen aus dem Entomologischen Verein in Bremen 26, 6-30.

NA (1994): Bienenweide auf stillgelegten Ackerflächen, Ergebnisse der Feldversuche 1992 und 1993 mit Tübinger Mischung. 156 pp.

Warncke, K.; Kullenberg, B. (1984): Synopsis of observations of *Andrena* and *Colletes cunicularius* males on *Ophrys* flowers (Orchidaceae). In: Acta Universitatis Upsaliensis Nova Acta Regiae Societatis Scientiarum Upsaliensis Series V C 3, 41-56.

Wussow, J.; Dix, V. (1977): Zur Hummelfauna (Hymenoptera. Bombidae) der mittleren Ostseeküste der DDR. Entomologische Nachrichten und Berichte 21 (8), 119-122.

Not found

Anonymous (1986): Pollination biology. In: Research resume, 23-28.

Jung, G. (1995): Wildbienen im Wald: Eine Fallstudie auf einer als Bannwald eingerichteten Wibke-Wurffläche an einem Fichtenstandort des Tübinger Schönbuchs im 4. und 5. Sukzessionsjahr. 99 pp.

Schenck (1876): [Title unknown.] In: Entomologisches Nachrichtenblatt 2, unpaginated.

Weller, C. (1986): Zusammenhänge zwischen Pheromonabgabe, Größe und Verhalten bei *Lasioglossum malachurum* Königinnen und Arbeiterinnen. 79 pp.
