# Supplementary material for: What evidence exists on wild bee trends in Germany? A systematic map
Source: Environ Evid. 2025 Jun 19;14:11. doi: 10.1186/s13750-025-00364-7 (PMC12178071; doi:10.1186/s13750-025-00364-7)
Supplement: Supplementary file 1 — Supplementary Material 1: Additional file S1. Search term for searches in Web of Science and Scopus. Additional file S2. Search record. Additional file S3. ROSES form for systematic maps. Additional file S4. R code and data. Additional file S5. Excluded full text records with reasons for exclusion. Additional file S6. Unretrievable full texts. Additional file S7. Study clusters. Additional file S8. Species List of Hesse and Saxony-Anhalt, German. [file 13750_2025_364_MOESM1_ESM.zip › Supplements Environ Evid (2025-05-15)/Mupepele_Hellwig_et_al_2025_S7_Study_clusters.pdf]

What evidence exists on wild-bee trends in Germany? A systematic map

Anne-Christine Mupepele, Niels Hellwig, Petra Dieker, Alexandra-Maria Klein

Supplement S7: Study clusters

Tab. S1: Clusters of multiple studies covering data collected in a common study context

Cluster name	Cluster characteristics / Common study context	Bibtex keys in database	Information on covariates (IDs of Covariates Table)	Further references with relevant data
Jena	Common data	Ebeling2012, Rzanny2012, Fornoff2017	741	-
Blüthgen, Saale Valley	Continued data	Blüthgen1925, Blüthgen1929, Blüthgen1937	747, 270, 271	-
Blüthgen	Continued data	Blüthgen1949, Blüthgen1951, Blüthgen1961a	571, 572, 573, 574, 724, 725, 726, 729	[S1]
van der Smissen	Continued data	Smissen1991, Smissen2010	712, 713, 714	[S2]
Leipzig (Bienitz)	Complementary data from multiple studies	Gerth2012, Hausotte2017	679	[S3–S5]
TERENO sites, Saxony-Anhalt	Complementary data from multiple studies	Creutzburg2016, Papanikolaou2017, Thompson2021	722, 413, 681	[39,49,S6]
Wolf, Marburg	Continued data	Wolf1985b, Wolf1994	740	-
Cölln, Gönnersdorf	Continued data	Cölln1990, Hembach1998 Cölln2005	125, 126, 458, 569	[S7,S8]
Markowsky, Berlin	Continued data	Markowsky1933, Markowsky1940, Markowsky1943	307,429, 326	[S9]
Haeseler, Steller Heide	Continued data	Haeseler2005, Haeseler2013	134, 605	-
Haeseler, Mellum Island	Continued data	Haeseler1978b, Haeseler1982, Haeseler1988	139, 140, 141, 142, 143, 527, 744, 745	[S10]
Wickl, Amberg-Sulzbach	Complementary data from multiple studies	Wickl1994b, Wickl1994c, Wickl2005, Wickl2009	629, 630, 322, 323, 324, 79	[S11–S13]

Tab. S1 *Continued*.

**Cluster name**	**Cluster characteristics / Common study context**	**Bibtex keys in database**	**Information on covariates (IDs of Covariates Table)**	**Further references with relevant data**
Hallmen, Frankfurt (Berger Hang)	Continued data	Hallmen1995, Hallmen1999, Hallmen2016	535, 485	[S14]
Lower Oder Valley	Continued data	Flügel2001, Flügel2009, Saure2016b	240, 543, 613	-
Westrich, Tübingen	Common data	Westrich1979, Westrich1980	626, 628	-
Checklist Thuringia	Continued data	Burger2001, Burger2002, Burger2003b, Burger2004a	697, 687, 701, 435	[S15]
Jacobi, *Megachile rotundata* in North Rhine-Westphalia	Continued data	Jacobi2002, Jacobi2006	93	-
Balles, Baden	Continued data	Balles1925, Balles1926, Balles1927a, Balles1927b, Balles1933, Balles1939, Balles1952	74, 75, 76, 703, 77, 643, 78	-

References

S1. Blüthgen P. Neues oder Wissenswertes über mitteleuropäische Aculeaten und Goldwespen III.. Bonn zool Beitr. 1954;5:139–55.

S2. van der Smissen J. Zweiter Beitrag zur Bienen- und Wespenfauna im südöstlichen Schleswig-Holstein und nordöstlichen Niedersachsen (Hymenoptera: Aculeata). Drosera. 1993;‘93:125–34.

S3. Krieger R. Ein Beitrag zur Kenntnis der Hymenopterenfauna des Königreichs Sachsen. Leipzig: Dürr; 1894.

S4. Müller H. Beiträge zur Kenntnis der Bienenfauna Sachsens. Mitt Dt Ent Ges. 1944;13:65–108.

S5. Müller H. Faunistisch-ökologische Untersuchungen auf den Bienitzwiesen bei Leipzig unter besonderer Berücksichtigung der Heuschrecken. Wiss Z Karl-Marx-Univ. 1954;Math.-naturw. Rh. 4:73–80.

S6. Hofmann S, Everaars J, Schweiger O, Frenzel M, Bannehr L, Cord AF. Modelling patterns of pollinator species richness and diversity using satellite image texture. PLoS ONE. 2017;12:e0185591.

S7. Cölln K. Nachtrag zu Dendrocopos 17 (1990): 109–117. Dendrocopos. 1991;18:152–3.

S8. Hembach J, Cölln K. Die Wildbienen (Hymenoptera, Apidae) von Gönnersdorf (Kr. Daun). Dendrocopos. 1993;20:170–99.

S9. Markowsky H. Einige seltenere Bienen aus der Umgebung Berlins, die ich im Jahre 1936 erbeutete. (Hym. Apid.). Mitt Dt Ent Ges. 1940;9:65–6.
S10. Drescher W. Die Hummelfauna der Mellum. Abh Naturwiss Verein Bremen. 1974;38:197–9.
S11. Wickl K-H. Bemerkenswerte Vorkommen von Bienen, Wespen und Ameisen in Sandgebieten der Oberpfalz (Hymenoptera Aculeata). Galathea. 1999;15:95–119.
S12. Wickl K-H. Wildbienen des Landkreises Amberg-Sulzbach (Oberpfalz) (Hymenoptera, Apidae). Galathea. 2000;16:33–53.
S13. Wickl K-H. Wildbienen der Region Obere Vils-Ehenbach (Lkr. Amberg-Sulzbach) Bestand, Gefährdung und Verluste der Apidae unter besonderer Berücksichtigung der im Boden in Aggregationen nistenden Arten (Hymenoptera: Apidae). Galathea. 2010;26:171–95.
S14. Hallmen M, Wolf H. Die Bienenfauna des Naturschutzgebietes "Am Berger Hang" im Osten von Frankfurt am Main. (Hymenoptera: Apidae). Hess Faunist Briefe. 1993;13:53–61.
S15. Burger F, Conrad D. Vierter Nachtrag zur Bienenfauna Thüringens (Hymenoptera, Apidae). Checklisten Thüringer Insekten. 2005;13:57–9.
