# Supplementary material for: What evidence exists on wild bee trends in Germany? A systematic map
Source: Environ Evid. 2025 Jun 19;14:11. doi: 10.1186/s13750-025-00364-7 (PMC12178071; doi:10.1186/s13750-025-00364-7)
Supplement: Supplementary file 1 — Supplementary Material 1: Additional file S1. Search term for searches in Web of Science and Scopus. Additional file S2. Search record. Additional file S3. ROSES form for systematic maps. Additional file S4. R code and data. Additional file S5. Excluded full text records with reasons for exclusion. Additional file S6. Unretrievable full texts. Additional file S7. Study clusters. Additional file S8. Species List of Hesse and Saxony-Anhalt, German. [file 13750_2025_364_MOESM1_ESM.zip › Supplements Environ Evid (2025-05-15)/Mupepele_Hellwig_et_al_2025_S4_RCode_Data/Review_Insect_decline_Supplement.html]

Mupepele\_Hellwig\_et\_al\_2025\_S4\_RCode\_Data


# Mupepele\_Hellwig\_et\_al\_2025\_S4\_RCode\_Data

#### Anne Mupepele & Niels Hellwig

#### 05/13/2025

Supplement S4 for the article: Mupepele, A-C; Hellwig, N; Dieker, P;
Klein, A-M (2025) What evidence exists on wild bee trends in Germany? A
systematic map

```
  library(knitr)
  knitr::opts_chunk$set(warning = FALSE, message = FALSE)
```

```
library(car) #for recoding data
library(RColorBrewer)
library(plotrix) #plot with broken x axis
library(DBI) #to connect to MariaDB
library(gplots) #for function balloonplot (graphically animated table)
library(dplyr)
library(DBI)
library(ggplot2)
library(stringr)
library(sf)
library(osmdata)
library(ggmap)
library(VennDiagram)
# library(gridExtra)
library(ggpubr)
```

# Data

```
studies <- read.csv("studies_2025_05.csv",fileEncoding="UTF-8",stringsAsFactors = FALSE)
covariates <- read.csv("covariates_2025_05.csv",fileEncoding="UTF-8",stringsAsFactors = FALSE)
data <- read.csv("data_2025_05.csv",fileEncoding="UTF-8",stringsAsFactors = FALSE)
```

# Fig 1 Roses Diagram

see pdf

# Fig 2 Studies per year

```
years_df <- plyr::count(studies, "year")
plot_years <- ggplot(years_df, aes(x = year, y = freq)) +
  geom_bar(position = "stack", stat = "identity", width = 0.7, colour = "gray60", fill = "gray60") + 
  labs(x = "Year", y = "Study count") +
  scale_x_continuous(expand = c(0,0), limits = c(1880, 2023), breaks = seq(1880, 2020, 10)) +
  scale_y_continuous(expand = c(0,0), limits = c(0, 20), breaks = seq(0, 20, 2)) +
  theme(panel.background = element_rect(fill = "white"),
        panel.border = element_rect(colour = "black", fill = NA),
        panel.spacing = unit(1, "lines"),
        plot.title = element_text(size = 13, face = "bold", margin = margin(10, 0, 0, 0)),
        plot.subtitle = element_text(size = 12, face = "italic", margin = margin(5, 0, 10, 0)),
        axis.title = element_text(size = 14, hjust = 0.5, vjust = 2),
        axis.text.x = element_text(size = 12, vjust = 0.5, colour = "black"),
        axis.text.y = element_text(size = 12, vjust = 0.5, colour = "black"),
        legend.position = "none")
plot_years
```

```
#ggsave("Fig2_StudiesPerYears.tiff", plot = plot_years, width = 16 * 0.5, height = 9 * 0.5, dpi = 500, compression = "lzw")
#ggsave("Fig2_StudiesPerYears.png", plot = plot_years, width = 16 * 0.5, height = 9 * 0.5, dpi = 500)
```

# Fig 3 Studies per journal

```
## Fig. 3: Journals x Year groups ----
tab_journals <- table(studies[studies$journal != "",]$journal)
tab_journals_ordered <- tab_journals[order(tab_journals, decreasing = T)]
tab5_journals <- tab_journals_ordered[tab_journals_ordered >= 5]
journals5_df <- data.frame(tab5_journals)

studies$year_group_25 <- floor(studies$year / 25) * 25
studies_sel <- studies[studies$journal != "",]
journals_x_year <- summarise(group_by(studies_sel, journal, year_group_25), n = n())
names(journals5_df) <- c("journal", "freq")
journals5_x_year_df <- plyr::join(journals5_df, journals_x_year, by = "journal")
journals5_x_year_df$journal <- factor(journals5_x_year_df$journal, levels = rev(levels(journals5_x_year_df$journal)))
journals5_x_year_df$year_group_25 <- factor(journals5_x_year_df$year_group_25)

plot_journals5_x_year <- ggplot(journals5_x_year_df, aes(x = journal, y = n)) +
  geom_bar(aes(fill = year_group_25), position = "stack", stat = "identity", width = 0.7, colour = "gray50") + 
  geom_text(aes(label = freq, y = freq + 0.3), hjust = -0.02) +
  # ggtitle("c") +
  labs(x = "Author names", y = "Study count", fill = "Year") +
  scale_y_continuous(expand = c(0,0), limits = c(0, 24), breaks = seq(0, 24, 1)) +
  scale_fill_manual(values = c("1875" = "white", "1900" = "gray80", "1925" = "gray60", "1950" = "gray40", "1975" = "gray20", "2000" = "gray0"), 
                    labels = c("until 1899", "1900-1924", "1925-1949", "1950-1974", "1975-1999", "from 2000")) +
  coord_flip() +
  theme(panel.background = element_rect(fill = "white"),
        panel.border = element_blank(),
        axis.line.y = element_line(colour = "black"),
        panel.spacing = unit(1, "lines"),
        plot.title = element_text(size = 13, face = "bold", margin = margin(10, 0, 0, 0)),
        plot.subtitle = element_text(size = 12, face = "italic", margin = margin(5, 0, 10, 0)),
        axis.title = element_blank(),
        axis.text.x = element_blank(),
        axis.text.y = element_text(size = 12, vjust = 0.5, colour = "black"),
        axis.ticks.x = element_blank(),
        legend.title = element_text(size = 12, face = "bold"),
        legend.text = element_text(size = 12),
        legend.position = c(0.87, 0.13),
        legend.background = element_rect(linetype = "solid", colour = "darkgrey"),
        legend.spacing.x = unit(0.2, "cm"))
plot_journals5_x_year
```

```
#ggsave("Fig3_journals5_per_year_group.tiff", plot = plot_journals5_x_year, width = 0.8 * 16, height = 0.8 * 9, dpi = 500, compression = "lzw")
#ggsave("Fig3_journals5_per_year_group.png", plot = plot_journals5_x_year, width = 0.8 * 16, height = 0.8 * 9, dpi = 500)
```

# Fig 4 Spatial distribution

Maps have been produced with QGIS. Preliminary steps to produce data
and shapefiles have been realized in the two subsequent R-Code chunks.
Note that the results of the getbb function depend on the detectability
of location names in the current version of OpenStreetMap.

```
## Fig. 4a: Map federal states ----
df_states <- table(covariates$federalstate) %>% as.data.frame()
df_states$Var1 <- as.character(df_states$Var1)
df_states <- rbind(df_states, c("Hamburg", 0))
df_states$NUTS_NAME <- c("Baden-Württemberg", "Bayern", "Berlin", "Brandenburg", "Bremen", "Hessen", "Mecklenburg-Vorpommern", "Niedersachsen",
                         "Nordrhein-Westfalen", "Rheinland-Pfalz", "Saarland", "Sachsen", "Sachsen-Anhalt", "Schleswig-Holstein", "Thüringen", "Hamburg")
df_states$Freq <- as.numeric(df_states$Freq)
# write.csv2(df_states, "freq_federalstates.csv", row.names = F, col.names = F)
```

```
## Fig. 4b: Map location points ----
covariates[covariates$area == "multiple",]$area <- ""
covariates[covariates$site == "multiple",]$site <- ""
covariates[covariates$site == "noSelection",]$site <- ""
covariates[covariates$site == "unspecified",]$site <- ""
location_names <- gsub(",$", "", str_trim(gsub("^\\,", "", do.call(paste, c(covariates[c("area", "site")], sep = ", ")))))

st_years <- NULL
locations <- NULL
locations_years <- data.frame(location = rep(NA, length(location_names)), year = rep(NA, length(location_names)))
for(i in 1:length(location_names)){
  curr_bb <- NULL
  tryCatch({
    curr_bb <- getbb(location_names[i])
    if(!is.null(curr_bb)){
      locations_years$year[i] <- covariates$start_year[i]
      st_years <- rbind(st_years, covariates$start_year[i])
    }
    centroid <- st_as_sf(st_centroid(st_as_sfc(st_bbox(st_as_sf(data.frame(t(curr_bb)), coords = c("x", "y"))))))
    st_crs(centroid) <- 4326
    locations_years$location[i] <- st_as_sf(centroid)
    locations <- rbind(locations, st_as_sf(centroid))
  }, error = function(msg){
    message(paste0("ERROR in ", i, ": ", msg))
  }, warning = function(msg){
    message(paste0("WARNING in ", i, ": ", msg))
  })
}
locations$year <- as.numeric(na.omit(locations_years$year))
# st_write(locations, "covariates_locations_years_v3.shp")
```

# Fig 5 Land-use categories

```
cov_sub <- covariates[!covariates$landuse_cluster%in%c('multiple','unspecified'),]
cov_sub$entries <- rep(1,nrow(cov_sub))
dt1 <- with(cov_sub,tapply(entries,list(landuse_cluster,federalstate),sum))
dt1[is.na(dt1)] <- 0
dt <- as.table(as.matrix(dt1))[order(rowSums(dt1),decreasing=T),]
#Figure number: #landuse
#cairo_pdf(file="Fig5_balloonplot_landuse_federalstate.pdf",width = 9,height =5)
balloonplot(dt,  main ="", xlab ="", ylab="", show.margins = F, cum.margins=F, dotsize = 6, dotcolor =  "#add8e6", text.size = .8,text.color="black", rowmar=3, colmar=2,colsrt=30,label.size=0.7,sorted=F)
```

```
#dev.off()
```

# Fig 6 Bee sampling methods

Arranging data format. Some covariate entries are providing data
based on several methods.

```
d <- unlist(sapply(1:nrow(covariates),function(i)paste(str_split(covariates$sampling_methods,"&|/")[[i]],covariates$start_year[i],covariates$wildbee_measure[i],covariates$covariate_id[i],sep="_")))
sampling_split <- data.frame(t(data.frame(str_split(d,"_"))),row.names=NULL)
colnames(sampling_split) <- c("sampling_method","start_year","wildbee_measure","covariate_id")

sampling_split$sampling_method <- 
     car::recode(str_trim(sampling_split$sampling_method),"
c('active catches','aerial netting','aerial netting along transects')='netting';
c('bowl trap','Bowl trap','pan trap','yellow trap')='pan trap';
c('collecting dead specimens')='dead specimen';
c('flight trap','Malaise trap','malaise trap','air eclector')='malaise trap';
c('flower observation','flower observations','flower observations along transects','flower observations of Anthericum liliago','flower observations of Resedea','flower ovservations','tree observation')='flower observation';
c('observation','occasional oberservation','occasional obersvation','occasional observation','photos','targeted search','nest observation')='observation';
c('emergence net','ground eclector','stem eclector')='emergence trap';
c('literature, private samples, museum samples','museum')='museum specimen';
c('pitfall trap')='pitfall trap';
c('transect walks','bird prey transect collection')='transect';
c('trap nests','traps','self-made nesting device')='trap nest'
")

sampling_split$sampling_method[sampling_split$sampling_method%in%c("pitfall trap","emergence trap","dead specimen","museum specimen","suction")] <- 'other'

table(sampling_split$sampling_method,sampling_split$wildbee_measure)
```

```
##                     
##                      abundance presence records species richness
##                             18               58                3
##   flower observation         3               10                1
##   malaise trap               1                8                4
##   netting                    8               40               25
##   observation               60              104               28
##   other                      3               11                8
##   pan trap                   2               12                6
##   transect                   1                6                2
##   trap nest                  6                5                1
##   unspecified               16               98               25
##                     
##                      species richness & abundance
##                                                 4
##   flower observation                           15
##   malaise trap                                 17
##   netting                                      63
##   observation                                  39
##   other                                         2
##   pan trap                                     22
##   transect                                      3
##   trap nest                                     4
##   unspecified                                   5
```

```
sampling_split$start_year <- as.numeric(sampling_split$start_year)
sampling_split$sampling_method <- factor(sampling_split$sampling_method,levels = names(sort(table(sampling_split$sampling_method),decreasing=T))[c(1:7,9:10,8)])
```

```
plot_sampling <- ggplot(data=droplevels(sampling_split[!sampling_split$sampling_method%in%c("","unspecified","multiple"),]), aes(x=start_year)) + 
     geom_histogram(bins=10,position="stack")+
     facet_wrap(~sampling_method, scales = "fixed",ncol=4,strip.position = "top")+
     labs(y = "Number of covariate entries", x = 'Sampling Start [year]')+
      theme_light() +
     scale_y_continuous(trans='log10')+
  theme(axis.text.x = element_text(size = 10),strip.text = element_text(color ="black", hjust = 0, size = 10),strip.background = element_rect(fill = "grey95", linetype = "solid",color = "black", linewidth = .2))
#ggsave("Fig6_plot_sampling.tiff", plot = plot_sampling, width = 7, height = 4, dpi = 500, compression = "lzw")
```

# Fig 7 Wild bee records are given as abundance presence records or species richness and abundance

```
wildbeerecords <- ggplot(data=sampling_split, aes(x=start_year)) + 
     geom_histogram(bins=10,position="stack")+
     facet_wrap(~wildbee_measure, scales = "fixed",ncol=2,strip.position = "top")+
     labs(y = "Number of covariate entries", x = 'Sampling Start [year]')+
      theme_light() +
     scale_y_continuous(trans='log10')+
  theme(axis.text.x = element_text(size = 10),strip.text = element_text(color ="black", hjust = 0, size = 10),strip.background = element_rect(fill = "grey95", linetype = "solid",color = "black", linewidth = .2))
#ggsave("Fig7_wildbeerecords.tiff", plot = wildbeerecords, width = 7, height = 4, dpi = 500, compression = "lzw")
```

# Fig 8 Number of entries in Covariates table per number of studies covered (in years)

```
covariates_yr <- covariates[c("start_year", "end_year")]
covariates_yr$duration <- as.numeric(covariates_yr$end_year) - as.numeric(covariates_yr$start_year)
covariates_yr_df <- plyr::count(covariates_yr, "duration")

plot_duration <- ggplot(covariates_yr_df, aes(x = duration, y = freq)) +
  geom_bar(position = "stack", stat = "identity", width = 0.7, colour = "gray60", fill = "gray60") + 
  labs(x = "Period covered by study (years)", y = "Number of entries in Covariates table") +
  scale_x_continuous(expand = c(0,0), limits = c(0, 162), breaks = seq(0, 160, 20)) +
  scale_y_continuous(expand = c(0,0), limits = c(0, 120), breaks = seq(0, 120, 20)) +
  theme(panel.background = element_rect(fill = "white"),
        panel.border = element_rect(colour = "black", fill = NA),
        panel.spacing = unit(1, "lines"),
        plot.title = element_text(size = 13, face = "bold", margin = margin(10, 0, 0, 0)),
        plot.subtitle = element_text(size = 12, face = "italic", margin = margin(5, 0, 10, 0)),
        axis.title = element_text(size = 14, hjust = 0.5, vjust = 2),
        axis.text.x = element_text(size = 12, vjust = 0.5, colour = "black"),
        axis.text.y = element_text(size = 12, vjust = 0.5, colour = "black"),
        legend.position = "none")
plot_duration
```

```
#ggsave("Fig8_duration.tiff", plot = plot_duration, width = 16 * 0.5, height = 9 * 0.5, dpi = 500, compression = "lzw")
#ggsave("Fig8_duration.png", plot = plot_duration, width = 16 * 0.5, height = 9 * 0.5, dpi = 500)
```

# Fig 9 Venn Diagramm

```
# Extract years
data$year <- as.numeric(sub(".*(\\d{4}).*", "\\1", data$time))

plots <- list()
for(state in c("Saxony-Anhalt", "Hesse")){
  data_sel <- data[data$federalstate == state,]
  yr_min <- min(data_sel$year)
  yr_max <- max(data_sel$year)
  state_abbr <- toupper(paste(substring(state, 
                                        c(1, nchar(state)), 
                                        c(1, nchar(state))), collapse = ""))
  
  # Separate sections for Venn diagram
  data_sel$venn_section <- NA
  data_sel[data_sel$year <= 1950,]$venn_section <- 1
  data_sel[data_sel$year > 1950 & data_sel$year <= 2000,]$venn_section <- 2
  data_sel[data_sel$year > 2000,]$venn_section <- 3
  table(data_sel$venn_section) # Number of data table entries per section
  
  venn_data <- list(time1 = sort(unique(data_sel[data_sel$venn_section == 1,]$bee_species)),
                    time2 = sort(unique(data_sel[data_sel$venn_section == 2,]$bee_species)),
                    time3 = sort(unique(data_sel[data_sel$venn_section == 3,]$bee_species)))
  
  # Venn diagram
  plots[[state]] <- venn.diagram(venn_data,
                                main = state,
                                category.names = c(paste0(yr_min, "–1950"), "1951–2000", paste0("2001–", yr_max)),
                                main.pos = c(0.5, 1.1), main.cex = 1.6, 
                                main.fontface = "bold", main.fontfamily = "Arial",
                                cat.dist = c(0.08, 0.08, 0.05), cat.cex = rep(1.6, 3), cex = rep(1.6, 7),
                                fill = c("#E69F00", "#56B4E9", "#009E73"),
                                width = 7 * 0.5, height = 7 * 0.5,
                                filename = NULL)
  
  # Venn diagram to file
  # venn.diagram(venn_data,
  #              main = state,
  #              category.names = c(paste0(yr_min, "–1950"), "1951–2000", paste0("2001–", yr_max)),
  #              main.pos = c(0.5, 1.1), main.cex = 1.6, 
  #              main.fontface = "bold", main.fontfamily = "Arial",
  #              cat.dist = c(0.08, 0.08, 0.05), cat.cex = rep(1.6, 3), cex = rep(1.6, 7),
  #              fill = c("#E69F00", "#56B4E9", "#009E73"),
  #              filename = paste0("Fig9_venn_", state_abbr, ".tif"))
}

# Venn diagram to display
ggarrange(plotlist = plots, ncol = 2, widths = rep(9 * 0.5, 2))
```

# S8 Supplement with species lists

```
data$year <- as.numeric(sub(".*(\\d{4}).*", "\\1", data$time))
data$time_period <- cut(data$year, 
                      breaks = c(-Inf, 1950, 2000, Inf), 
                      labels = c("prior to 1950", "1950 to 2000", "after 2000"))

table_Hesse <- table(data$bee_species[data$federalstate == "Hesse"], data$time_period[data$federalstate == "Hesse"])
table_Hesse[table_Hesse>1] <- 1
table_SaxonyAnhalt <- table(data$bee_species[data$federalstate == "Saxony-Anhalt"], data$time_period[data$federalstate == "Saxony-Anhalt"])
table_SaxonyAnhalt[table_SaxonyAnhalt>1] <- 1
#write.csv(table_Hesse,"specieslist_Hesse.csv",fileEncoding="UTF-8") 
#write.csv(table_SaxonyAnhalt,"specieslist_SaxonyAnhalt.csv",fileEncoding="UTF-8")
```
