# Supplementary figures and images for: What evidence exists on wild bee trends in Germany? A systematic map
Source: Environ Evid. 2025 Jun 19;14:11. doi: 10.1186/s13750-025-00364-7 (PMC12178071; doi:10.1186/s13750-025-00364-7)

Searching

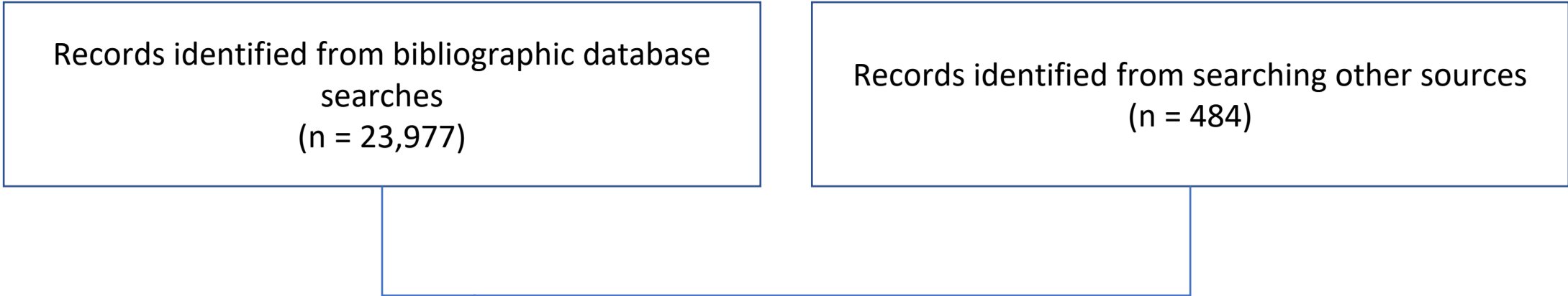

Screening

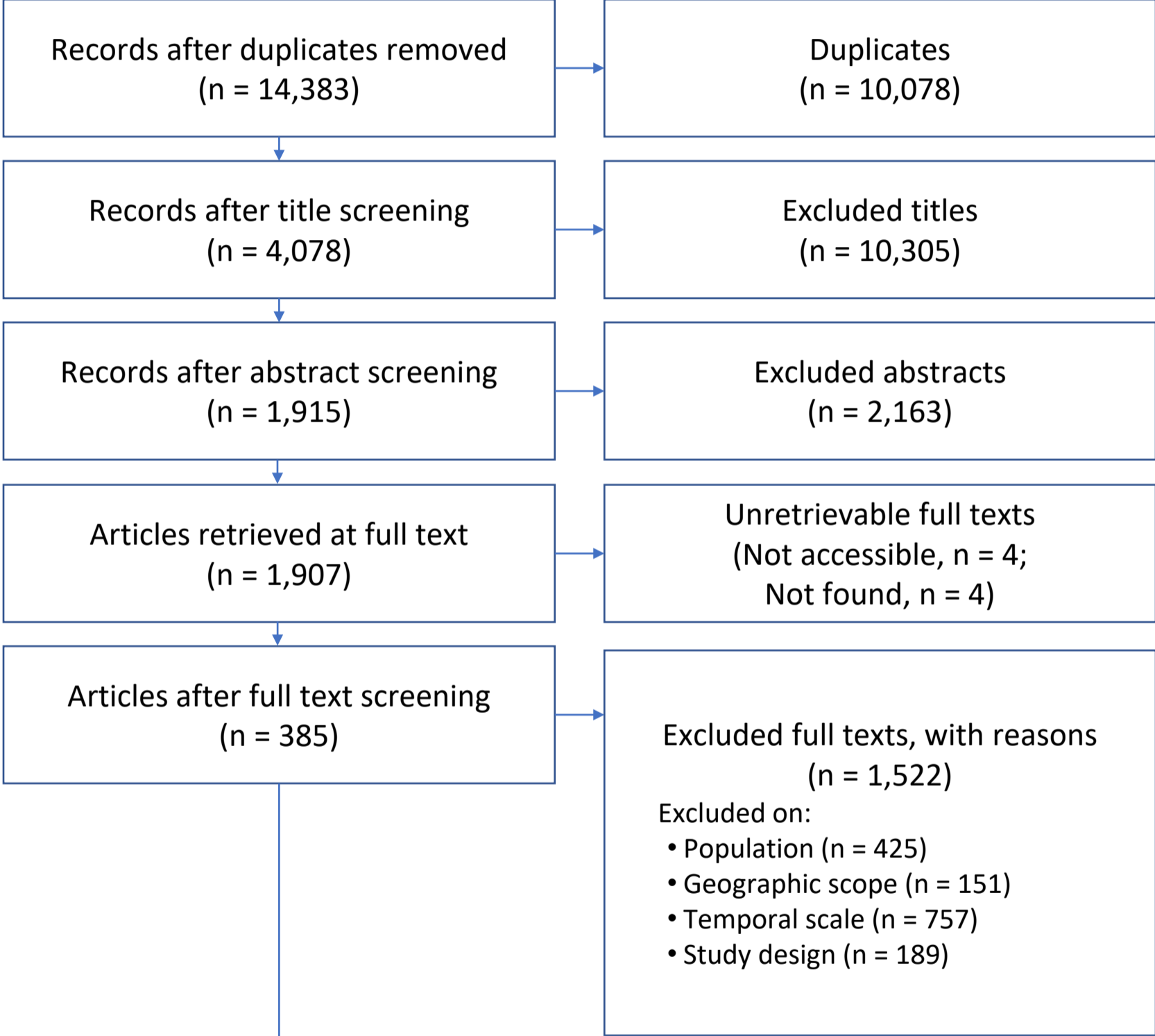

Synthesis

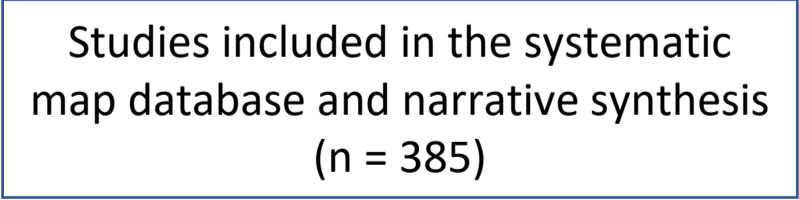

Supplement: Supplementary file 1 — Supplementary Material 1: Additional file S1. Search term for searches in Web of Science and Scopus. Additional file S2. Search record. Additional file S3. ROSES form for systematic maps. Additional file S4. R code and data. Additional file S5. Excluded full text records with reasons for exclusion. Additional file S6. Unretrievable full texts. Additional file S7. Study clusters. Additional file S8. Species List of Hesse and Saxony-Anhalt, German. [file 13750_2025_364_MOESM1_ESM.zip › Supplements Environ Evid (2025-05-15)/Mupepele_Hellwig_et_al_2025_S3_ROSES_flow_diagram.pdf]
